# Highly prevalent MDR, frequently carrying virulence genes and antimicrobial resistance genes in Salmonella enterica serovar 4,[5],12:i:- isolates from Guizhou Province, China

**DOI:** 10.1371/journal.pone.0266443

**Published:** 2022-05-19

**Authors:** Li Long, Lv You, Dan Wang, Ming Wang, Junhua Wang, Guihuan Bai, Jianhua Li, Xiaoyu Wei, Shijun Li

**Affiliations:** 1 Laboratory of Bacterial Disease, Experimental Center, Guizhou Provincial Center for Disease Control and Prevention, Guiyang, People’s Republic of China; 2 Institute of Communicable Disease Control and Prevention, Guizhou Provincial Center for Disease Control and Prevention, Guiyang, People’s Republic of China; 3 School of Public Health, the Key Laboratory of Environmental Pollution Monitoring and Disease Control, Ministry of Education, Guizhou Medical University, Guiyang, China; 4 Tongren City Center for Disease Control and Prevention, Tongren, People’s Republic of China; Cornell University, UNITED STATES

## Abstract

*Salmonella enterica* serovar 4,[5],12:i:-, a monophasic variant of *Salmonella* Typhimurium lacking the phase 2 flagellin, is one of the common serotypes causing *Salmonellosis* worldwide. However, information on *Salmonella* serovar 4,[5],12:i:- from Guizhou Province has lacked so far. This study aimed to investigate the antimicrobial resistance, the presence of antimicrobial resistance genes and virulence genes, and characterize the MLST genotypes of *Salmonella* serovar 4,[5],12:i:- isolates from Guizhou province, China. We collected 363 non-typhoid *Salmonella* (NTS) isolates of Guizhou from 2013 to 2018. Biochemical identification, serogroups testing, and specific multiplex polymerase chain reaction (mPCR) assay were conducted to identify *Salmonella* 4,[5],12:i:- isolates. Isolates were determined the antimicrobial resistance by the micro broth dilution method, detected the presence of antimicrobial resistance genes and virulence genes by PCR, and examined the molecular genotyping by Multilocus sequence typing (MLST). Eighty-seven *Salmonella* 4,[5],12:i:- isolates were detected, accounting for 23.9% (87/363) of the total NTS isolates. All *Salmonella* 4,[5],12:i:- isolates showed highly resistant to sulfaoxazole (93.1%), streptomycin (90.8%), ampicillin (88.5%), tetracycline (86.2%) and doxycycline (86.2%). A high proportion (94.2%) of multi-drug resistance (MDR) isolates were found. Most (83.9%) *Salmonella* 4,[5],12:i:- isolates carried four antimicrobial resistance genes, especially *bla*_*TEM-1*_, *strA-strB*, *sul2*, and *tetB* genes. *Salmonella* 4,[5],12:i:- isolates showed a high rate of *invA*, *sseL*, *mgtC*, *siiE*, *sopB*, *gipA*, *gtgB*, *sspH1*, and *sspH2* (72.4%~98.9%). On the contrary, none of the isolates were detected the *spvC* and *pefA* genes. MLST analysis revealed three sequence types (STs), and ST34 (97.7%) was the dominant sequence type. This study is the first report of *Salmonella* 4,[5],12:i:- in humans from Guizhou province, China. The data might be useful for rational antimicrobial usage against *Salmonella* 4,[5],12:i:- infections, risk management, and public health strategies in Guizhou.

## Introduction

Non-typhoid *Salmonella* (NTS) is one of the most common causes of human infectious diarrheal diseases and causes a severe disease burden [1]. In the late 1980s, a *Salmonella* serovar 4,[5],12:i:-, closely linked to the antigenic structure and genetic characterization of *Salmonella* Typhimurium, was first identified from poultry in Portugal [2]. Since then, this serotype has been increasingly spread around the world and has caused more significant outbreaks in Luxembourg and Italy [3, 4].

Multi-drug resistance (MDR) has been increased all worldwide that is considered a public health threat. Several recent investigations reported the emergence of multidrug-resistant bacterial pathogens from different origins including humans, birds, fish, and cattles that increase the need for routine application of the antimicrobial susceptibility testing to detect the antibiotic of choice as well as the screening of the emerging MDR strains [5–8]. The rapid dissemination of *Salmonella* 4,[5],12:i:- is related to the increasing MDR, which is a significant problem of public health, and may represent the advantage of its pathogenesis [9, 10]. Significantly, the horizontal transfer of resistance genes mediated by mobile genetic elements such as transposons and plasmids may enhance the survival adaptability of this serotype [11].

*Salmonella* infects the host by first attaching to the host tissue and then invading the host cells through virulence factors, mainly including *Salmonella* pathogenicity island (SPI), plasmid virulence, prophage virulence and cell swelling toxins [12]. SpI-1 is necessary to invade of host non-phagocytes, and plays an essential role in *salmonella* invasion of macrophages and intestinal epithelial cells [13]. SpI-2 encodes a type III secretory system associated with systemic infection. It allows *Salmonella* to survive inside the macrophage and it facilitates spreading through the host body [13]. *Salmonella* plasmid virulence genes enhance the ability to spread and proliferate in the host, and most of them are associated with extra-intestinal infection in humans and animals [12, 13]. In addition, previous investigations indicated that *Salmonella* 4,[5],12:i:- serotype was more virulent than other serotypes [14]. The presence of multiple virulence determinants, along with the formation of biofilm, enables *Salmonella* 4,[5],12:i:- to infect humans and results in disease or death [14, 15].

*Salmonella* 4,[5],12:i:- is a monophasic serotype due to its lack of phase 2 flagellar antigen expression [10]. Several distinct molecular subtyping methods, including phage typing, multilocus sequence typing (MLST), pulsed-field gel electrophoresis (PFGE), and whole genome sequencing (WGS) were used to identify *Salmonella* 4,[5],12:i:–isolates [16–18]. Studies indicated that *Salmonella* 4,[5],12:i:- isolates belong to multiple clonal lines, which evolved from different *Salmonella* Typhimurium clonal ancestors through different genetic events [19, 20].

In Guizhou, the Southwest of China, *NTS* was one of the primary pathogens causing infectious diarrhea, and the distribution of serotypes was diverse [21, 22]. However, information on *Salmonella* serovar 4,[5],12:i:- from Guizhou is lacking. To provide a better understanding of the characterization *Salmonella* 4,[5],12:i:-, we characterized the antimicrobial resistance, the presence of antimicrobial resistance genes and virulence genes, and the genetic characterization of *Salmonella* 4,[5],12:i:- isolates in Guizhou from 2013 to 2018.

## Materials and methods

### Ethics statement

This study was reviewed and approved by the Ethics Review Committee of Guizhou Provincial Center for Disease Control and Prevention. All data were analyzed anonymously.

### Bacterial isolates and identification

A total of 363 *Salmonella* isolates were collected from nine different cities between 2013 and 2018 in Guizhou, including Anshun (n = 54), Bijie (n = 10), Guiyang (n = 76), Liupanshui (n = 10), Qiandongnan (n = 22), Qiannan (n = 19), Qianxinan (n = 12), Tongren (n = 97), and Zunyi (n = 63), and they were all obtained from clinical patients. Most isolates were available from stool samples of outpatients, and the source of a few isolates was unknown. The obtained samples were inoculated into selenite brilliant green sulfa enrichment (SBG), cultured at 36°C ± 1°C for 6–8 h (Youkang Biological, China). The enriched bacteria solution was inoculated on the *Salmonella* chromogenic medium, incubated at 36°Cfor 18–24 h (CHROMagar, France). Pale purple or purple colonies were selected and inoculated onto krebs disaccharide iron medium (KIA) and motility indol urea iron medium (MIU) at 36°C for 18–24 h (Cyclokay Biological, China). The isolates were systematically identified by API20E identification kits (Biomerieux, France). According to the White- Kaufmann- Le Minor Scheme [23], the confirmed *Salmonella* isolates were serotyped by slide agglutination test for O and H antigens (SSI, Denmark). Flagellar induction testing was performed on the isolates with antigenic formula (4,[5],12:i:-). If induction testing showed negative for H2 phase, the flagellar antigen gene (*fljB*) and IS200 fragment (*fljB*-*fljA*) of H2 phase were further detected by a multiple PCR as described by Tennant et al [24]. Primers used to amplify *fljB* and *fljB-fljA* genes were listed in S1 Table. Bacterial DNA was extracted by the boiled lysis method. The supernatant was taken as DNA template and stored at -80°C for use. The multiple PCR reaction conditions were as follows: initial denaturation at 95°C for 2 min, 30 cycles of denaturation at 95°C for 30 s, annealing at 58°C for 30 s, extension at 72°C for 90 s, and a final delay at 72°C for 10 min.

### Antimicrobial susceptibility test

Antimicrobial susceptibility was evaluated by micro broth dilution method with ten classes 16 antimicrobials (Xingbai Biological, China), including: Penicillin (Ampicillin), Phenicols (Chloramphenicol), Aminoglycosides (Streptomycin, Gentamicin), Carbapenems (Imipenem), β-lactamase inhibitor (Amoxicillin/clavulanic acid), Cephems (Cefoxitin, Ceftriaxone, Cefepime), Sulfonamides (Sulfamethoxazole, Trimethoprim /sulfamethoxazole), Tetracyclines (Tetracycline, Doxycycline), Quinolones and Fluoroquinolones (Nalidixic acid, Ciprofloxacine), Macrolides (Azithromycin). Escherichia coli ATCC 25922 was used as a control strain. The breakpoints for antimicrobials followed interpretive standards provided by Clinical Laboratory Standards Institute guidelines [25]. The phenotypic resistance profiles were classified into MDR, XDR, and PDR as described by Magiorakos et al [26].

### Detection of antimicrobial resistance genes

Genes coding for resistance to β-lactamase (*bla*_*TEM*_, *bla*_*OXA-1*_, *bla*_*CTX-M*_), phenicols (*floR*, *cmlA1*), aminoglycosides (*aac (3)-IV*, *strA-strB*, *aadA2*), sulfonamides (*sul2*), and tetracyclines (*tetB*) were evaluated by PCR using primers and conditions as previously described [27–29]. The reaction volume was 20 μl. Primers used to amplify the antimicrobial resistance genes and PCR reaction conditions in this study were listed in S2 Table. The agar gel electrophoresis was carried out to separate the obtained PCR products using 1.0% agarose, followed by photographing the gel. All positive PCR products of *bla*_*TEM*_ and *bla*_*CTX-M*_ genes were sequenced and aligned with the National Centre for Biotechnology Information (NCBI) database sequences using the BLAST program to identify resistance gene subtypes. The correlation between phenotypic and genotypic was performed.

### Detection of virulence genes

Fourteen virulence genes were detected by PCR. These virulence genes are related to the presence of Salmonella pathogenicity island (*invA*, *sseL*, *mgtC*, *siiE*, *sopB*), prophages (*gipA*, *gtgB*, *sopE*, *sspH1*, *sspH2*), and plasmids (*spvB*, *spvC*, *spvR*, *pefA*). The primer sequences as previously described [13, 30, 31] were listed in S3 Table. The PCR reaction conditions were as follows: initial denaturation at 94°C for 5 min, 28 cycles of denaturation at 94°C for 30 s, annealing at 58°C for 30 s, extension at 72°C for 1min, and a final delay at 72°C for 5 min. The PCR products were analyzed by electrophoresis and visualized under ultraviolet light.

### Multilocus sequence typing (MLST)

MLST typing was executed for all *Salmonella* 4,[5],12:i:- isolates based on seven housekeeping genes, including *thrA*, *purE*, *sucA*, *hisD*, *hemD*, a*roC* and *dnaN*. Primers used to amplify the seven housekeeping genes in this study were listed in S4 Table. The amplification conditions were as follows: initial denaturation at 94°C for 5min, 30 cycles of denaturation at 94°C for 30 S, annealing at 56°C for 1 min, extension at 72°C for 1 min, and a final delay at 72°C for 10 min. Alleles and ST types of isolates were obtained from the *Salmonella* database on the PubMLST website. The phylogenetic tree was constructed using BioNumerics 8.0 software (Applied-Maths, Belgium).

### Statistical analysis

We used Cohen’s kappa coefficient to assess the correlation between phenotypic and genotypic resistance. The agreement, as expressed by the kappa coefficient, was interpreted as follows: values ≤ 0 as indicating no agreement and 0.01–0.20 as none to slight, 0.21–0.40 as fair, 0.41–0.60 as moderate, 0.61–0.80 as substantial, and 0.81–1.00 as almost perfect agreement [32]. All *P* values were two-tailed, and the level of statistical significance was specified as 0.05. Statistical analyses were performed using version 26.0 SPSS statistical software.

## Results

### Phenotypic characteristics of the recovered *Samonella* isolates

**Fig 1:**
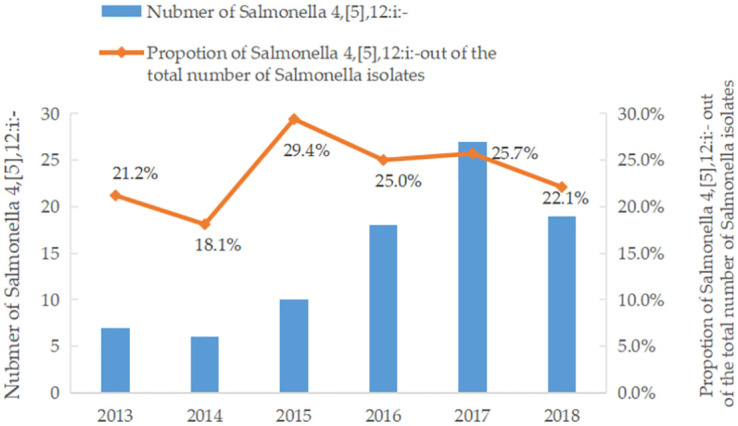
Detection of *Salmonella* 4,[5],12:i:- isolates from Guizhou, 2013–2018.

As described in the methods section, samples were enriched using SBG. A single colony purple or pale purple colony was observed on the *Salmonella* chromogenic medium. Biochemical results for *Salmonella* on KIA and MIU were slant (K), butt (A), H_2_S (+), gas (+), dynamic (+), indole (-), urea (-). After the multiple PCR detection, all *Salmonella* Typhimurium isolates produced two amplification bands (1 000 bp and 1 389 bp), specific to *Salmonella* Typhimurium. However, the *Salmonella* 4,[5],12:i:- isolates produced only one 1 000 bp amplification band (S1 Fig). Among the 363 NTS isolates, 23.9% (87/363) were confirmed as *Salmonella* 4,[5],12:i:-. They were distributed in seven of the nine cities in Guizhou, which were Anshun (n = 10), Guiyang (n = 14), Liupanshui (n = 2), Qiandongnan (n = 3), Qiannan (n = 2), Tongren (n = 37), and Zunyi (n = 20), respectively. The prevalence of *Salmonella* 4,[5],12:i:- ranged between 18.1% to 29.4% in 2013–2018 with the highest prevalence in 2015 (Fig 1).

### Antimicrobial resistance

Antimicrobial resistance testing showed that *Salmonella* 4,[5],12:i:- isolates were shown to be the most resistant to sulfaoxazole (93.1%), followed by streptomycin (90.8%), ampicillin (88.5%), tetracycline (86.2%), and doxycycline (86.2%). Furthermore, *Salmonella* 4,[5],12:i:- isolates showed resistance to chloramphenicol (42.5%), trimethoprim /sulfamethoxazole (35.6%), nalidixic acid (34.5%), and amoxicillin/clavulanic acid (31.0%), respectively (Table 1). The resistance to ciprofloxacin was 13.8%. However, 35.6% of isolates showed decreased sensitivity to ciprofloxacin (MIC≥0.12 μg/mL). Notably, *Salmonella* 4,[5],12:i:- isolates showed resistance to imipenem and azithromycin in this study. More importantly, three isolates were co-resistant to ciprofloxacin, the third and fourth-generation cephalosporins, and azithromycin.

**Table 1:** Antimicrobial resistance of 87 *Salmonella* 4,[5],12:i:- isolates.

Antimicrobial classes	Antimicrobial agents	Resistant		Intermediate		Susceptible	
		No.	%	No.	%	No.	%
Penicillin	Ampicillin (AMP)	77	88.5	0	0.0	10	11.5
Phenicols	Chloramphenicol (CHL)	37	42.5	16	18.4	34	39.1
Aminoglycosides	Streptomycin (STR)	79	90.8	0	0.0	8	9.2
	Gentamicin (GEN)	16	18.4	4	4.6	67	77.0
Carbapenems	Imipenem (IMP)	1	1.1	0	0.0	86	98.9
**β**-lactamase inhibitor	Amoxicillin/clavulanic acid (AMC)	27	31.0	11	12.6	49	56.3
Cephems	Cefoxitin (FOX)	1	1.1	3	3.4	83	95.4
	Ceftriaxone (CRO)	17	19.5	0	0.0	70	80.5
	Cefepime (FEP)	13	14.9	3	3.4	71	81.6
Sulfonamides	Sulfamethoxazole (SOX)	81	93.1	0	0.0	6	6.9
	Trimethoprim /sulfamethoxazole (SXT)	31	35.6	0	0.0	56	64.4
Tetracyclines	Tetracycline (TCR)	75	86.2	0	0.0	12	13.8
	Doxycycline (DOX)	75	86.2	7	8.0	5	5.7
Quinolones and Fluoroquinolones	Nalidixic acid (NAL)	30	34.5	0	0.0	57	65.5
	Ciprofloxacine (CIP)	12	13.8	31	35.6	4	50.6
Macrolides	Azithromycin (AZM)	5	5.7	0	0.0	82	94.3

CIP (ciprofloxacin), STR (streptomycin), AMP (ampicillin), CHL (chloramphenicol), SOX (sulfaisoxazole), SXT (trimethoprim sulfamethoxazole), NAL (nalidixic), AMC (amoxicillin / clavulanate potassium), CRO (ceftriaxone), DOX (doxycycline), GEN (gentamicin), AZM (azithromycin), TCY (tetracycline), FOX (cefxitin), FEP (cefepime), IMP (imipenem).

A percentage of 98.9% (86/87) isolates were resistant to at least one antimicrobial agent. Most (62.0%) isolates were resistant to 5–7 among the 16 antimicrobial agents tested. Notably, 12 isolates (13.8%) were resistant to ten or more antimicrobial agents, among which two isolates were resistant to 13 antimicrobial agents in 2016–2017. A total of 89.6% (78/87) of the isolates were MDR. Four isolates (4.5%) showed XDR (Table 2). PDR isolates were not observed. Fourty-five antimicrobial resistance profiles were observed, of which AMP+STR+SOX+TCR+ DOX+AMC (16.1%,14/87) and AMP+STR+SOX+TCR+DOX (14.9%, 14/87) were the predominant antimicrobial resistance profiles.

**Table 2:** Antimicrobial resistance profile of *Salmonella* 4,[5],12:i:- isolates.

No. of isolates	%	Type of resistance	antimicrobial resistance profile	No. of isolates	%
4	4.5	R	STR	1	1.1
			NAL	2	2.3
			TCR-DOX	1	1.1
78	89.6	MDR	AMP-STR-SOX-TCR-DOX-AMC	14	16.1
			AMP-STR-SOX-TCR-DOX	13	14.9
			AMP-CHL-STR-SOX-NAL-SXT	5	5.7
			AMP-CHL-STR-SOX-TCR-DOX-SXT	4	4.6
			AMP-STR-SOX-TCR-DOX-CRO-FEP	3	3.4
			AMP-CHL-STR-SOX-TCR-DOX-AMC	2	2.3
			AMP-CHL-STR-SOX-TCR-DOX-SXT-AMC	2	2.3
			AMP-CHL-STR-SOX-TCR-DOX-GEN-SXT	2	2.3
			AMP-CHL-STR-SOX-TCR-NAL-DOX-GEN-CIP-SXT-AMC	2	2.3
			STR-SOX-TCR-DOX	2	2.3
			AMP-CHL-STR-SOX-TCR-DOX	1	1.1
			AMP-CHL-STR-SOX-TCR-DOX-CRO-FEP	1	1.1
			AMP-CHL-STR-SOX-TCR-DOX-GEN-CIP-SXT-AZM	1	1.1
			AMP-CHL-STR-SOX-TCR-DOX-GEN-CRO	1	1.1
			AMP-CHL-STR-SOX-TCR-NAL-CIP-SXT	1	1.1
			AMP-CHL-STR-SOX-TCR-NAL-DOX-CIP-CRO-FEP	1	1.1
			AMP-CHL-STR-SOX-TCR-NAL-DOX-CIP-SXT	1	1.1
			AMP-CHL-STR-SOX-TCR-NAL-DOX-CRO	1	1.1
			AMP-CHL-STR-SOX-TCR-NAL-DOX-GEN-CIP-SXT	1	1.1
			AMP-CHL-STR-SOX-TCR-NAL-DOX-GEN-CIP-SXT-CRO-FEP	1	1.1
			AMP-CHL-STR-SOX-TCR-NAL-DOX-GEN-SXT	1	1.1
			AMP-CHL-STR-SOX-TCR-NAL-DOX-GEN-SXT-AMC	1	1.1
			AMP-CHL-SOX-NAL-AMC	1	1.1
			AMP-CHL-SOX-TCR-NAL-DOX-CIP	1	1.1
			AMP-STR-SOX	1	1.1
			AMP-STR-SOX-TCR	1	1.1
			AMP-STR-SOX-TCR-DOX-AMC-CRO-FEP	1	1.1
			AMP-STR-SOX-TCR-DOX-AMC-IMP	1	1.1
			AMP-STR-SOX-TCR-DOX-CRO	1	1.1
			AMP-STR-SOX-TCR-DOX-SXT-AMC	1	1.1
			AMP-STR-SOX-TCR-DOX-SXT-AMC-CRO-PEF	1	1.1
			AMP-STR-SOX-TCR-DOX-GEN-CIP-SXT-AZM-CRO-PEF	1	1.1
			AMP-STR-SOX-TCR-NAL-DOX-AMC	1	1.1
			AMP-STR-SOX-TCR-NAL-DOX-CIP	1	1.1
			AMP-SOX-TCR-NAL-DOX	1	1.1
			AMP-TCR-DOX-CRO-FEP	1	1.1
			CHL-STR-SOX-NAL-GEN-SXT	1	1.1
			CHL-STR-SOX-TCY-NAL-DOX-SXT	1	1.1
			STR-SOX-TCY-NAL-DOX-SXT	1	1.1
4	4.5	XDR	AMP-CHL-STR-SOX-TCR-NAL-DOX-GEN-CIP-SXT-AZM-CRO-FEP	2	2.3
			AMP-CHL-STR-SOX-TCR-NAL-DOX-SXT-AMC-CRO-FEP	1	1.1
			AMP-CHL-STR-SOX-TCR-NAL-DOX-GEN-AMC-CRO-FOX	1	1.1

CIP (ciprofloxacin), STR (streptomycin), AMP (ampicillin), CHL (chloramphenicol), SOX (sulfaisoxazole), SXT (trimethoprim sulfamethoxazole), NAL (nalidixic), AMC (amoxicillin / clavulanate potassium), CRO (ceftriaxone), DOX (doxycycline), GEN (gentamicin), AZM (azithromycin), TCY (tetracycline), FOX (cefxitin), FEP (cefepime), IMP (imipenem).

### Antimicrobial resistance genes distribution

A great majority (83.9%) of *Salmonella* 4,[5],12:i:- isolates contained at least four antimicrobial resistance genes. As regards antimicrobial resistance genes, genes more often detected were *tetB* (94.2%), *strA-strB* (93.1%), *sul2* (91.9%) and *bla*_*TEM-1*_ (74.7%). The existance of other resistance genes including *bla*_*OXA-1*_, *bla*_*CTX-M*_, *aac (3)-IV*, *aadA2*, *cmlA1*, and *floR* were 5.7%, 13.8%, 5.7%, 31.0%, 30.0%, and 20.2%, respectively. Among the positive isolates to β-lactamase resistance gene, nine isolates contained two β-lactamase genes (*bla*_*TEM-1*_*/bla*_*CTX-M*_, *bla*_*TEM-1*_*/bla*_*OXA-1*_), and one isolate contained *bla*_*TEM-1*_, *bla*_*CTX-M*_ and *bla*_*OXA-1*_ genes. Sequencing of the bla_CTX-M_ genes revealed presence of various types, including *bla*_*CTX-M-55*_ (58.3%, 7/12), *bla*_*CTX-M-65*_ (12.7%, 2/12), *bla*_*CTX-M-14*_ (8.3%, 1/12), *bla*_*CTX-M-15*_ (8.3%, 1/12), and *bla*_*CTX-M-27*_ (8.3%, 1/12) (S2 Fig).

### The correlation between the phenotypic and genotypic MDR profiles

**Fig 2:**
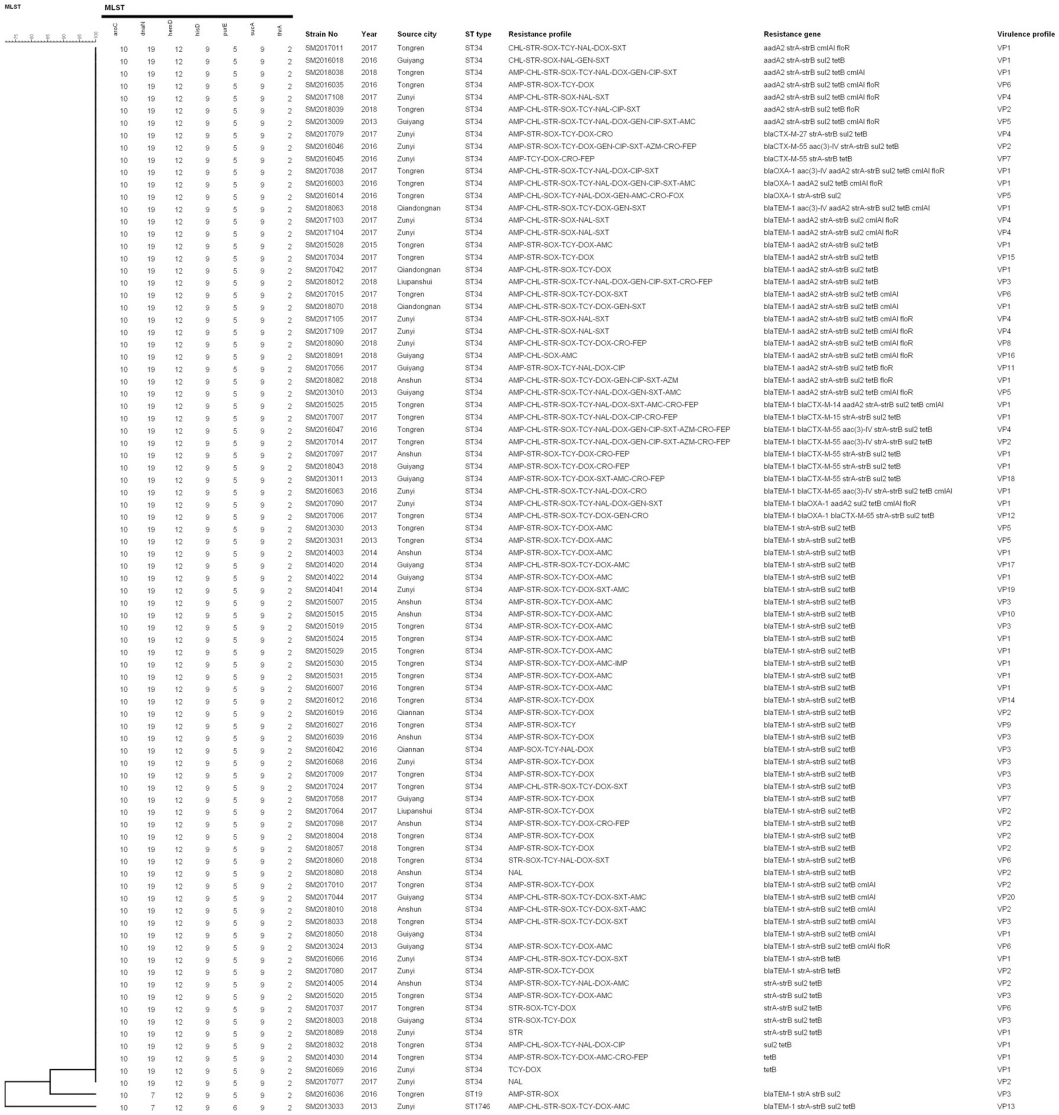
MLST clustering tree of the 87 *Salmonella* 4,[5],12:i:- isolates in Guizhou from 2013 to 2018 with the antimicrobial resistance profile, antimicrobial resistance genes, and virulence gene profile.

Our findings revealed that 12.6% (11/87) isolates were MDR to five antimicrobial classes (AMP, STR, SOX, TCR, DOX, AMC) and harbored *bla*_*TEM-1*_, *strA-strB*, *sul2*, *tetB*. Nine (10.3%) MDR isolates to four antimicrobial classes (AMP, STR, SOX, TCR, DOX) and harbored *bla*_*TEM-1*_, *strA-strB*, *sul2*, *tetB*. Besides, two (2.3%) XDR isolates to eight antimicrobial classes (AMP, CHL, STR, GEN, SOX, TCR, DOX, NAL, CIP, AMC, AZM, CRO, FEP) and harbored *bla*_*TEM-1*_, *bla*_*CTX-M-55*_, *aac(3) -IV*, *strA-strB*, *sul2*, *tetB* (Fig 2). The kappa correlation between phenotypic and genotypic resistance was showed in Table 3. Cohen’s kappa was the highest for *bla*_*CTX-M*_ vs CRO (Kappa = 0.794) and *bla*_*CTX-M*_ vs FEP (Kappa = 0.673), followed by *cmlA1* (Kappa = 0.509) vs CHL, *strA-strB* vs STR (Kappa = 0.418), and *tetB* vs TCY (Kappa = 0.388) (Table 3). In general, a certain correlation was seen between the antimicrobial phenotypes with genotypes.

**Table 3:** The correlation between the phenotypic and genotypic of *Salmonella* 4,[5],12:i:- isolates.

Antimicrobial resistance genes vs.antimicrobials	Gene (+)		Gene (-)			
	Phenotype (+)	Phenotype (-)	Phenotype (+)	Phenotype (-)	Kappa	*P*
*bla*_*TEM-1*_ vs AMP	63	2	15	7	0.357	<0.001
*bla*_*TEM-1*_ vs AMC	22	43	6	16	0.042	0.568
*bla*_*TEM-1*_ vs CRO	12	53	5	17	-0.025	0.663
*bla*_*TEM-1*_ vs FOX	0	65	1	21	-0.023	0.084
*bla*_*TEM-1*_ vs FEP	10	55	3	19	0.010	0.842
*bla*_*OXA-1*_ vs AMP	5	0	72	10	0.009	0.407
*bla*_*OXA-1*_ vs AMC	2	3	26	56	0.026	0.700
*bla*_*OXA-1*_ vs CRO	2	3	15	67	0.102	0.235
*bla*_*OXA-1*_ vs FOX	1	4	0	82	0.320	<0.001
*bla*_*OXA-1*_ vs FEP	0	5	13	69	-0.091	0.334
*bla*_*CTX-M*_ vs AMP	12	0	65	10	0.041	0.179
*bla*_*CTX-M*_ vs AMC	2	10	26	49	-0.115	0.215
*bla*_*CTX-M*_ vs CRO	12	0	5	70	0.794	<0.001
*bla*_*CTX-M*_ vs FOX	0	12	1	74	-0.020	0.681
*bla*_*CTX-M*_ vs FEP	9	3	4	71	0.673	<0.001
*cmlA1* vs CHL	22	4	15	46	0.509	<0.001
*floR* vs CHL	15	3	22	47	0.370	<0.001
*aac (3)-IV* vs STR	5	0	79	3	0.005	0.631
*aac (3)-IV* vs GEN	3	2	11	70	0.313	0.001
*aadA2* vs STR	27	0	53	7	0.076	0.064
*aadA2* vs GEN	10	17	5	55	0.327	0.001
*strA-strB* vs STR	77	4	3	3	0.418	<0.001
*strA-strB* vs GEN	14	67	2	4	-0.028	0.288
*sul2* vs SOX	76	4	4	3	0.321	0.003
*sul2* vs STX	32	48	2	5	0.013	0.765
*tetB* vs TCY	74	8	1	4	0.388	<0.001
*tetB* vs DOX	72	10	1	4	0.336	<0.001

### Virulence genes distribution

All *Salmonella* 4,[5],12:i:- isolates carried *invA*, *siiE* and *sopB* genes. The existence of other virulence genes including *sseL*, *mgtC*, *gipA*, *gtgB*, *sspH1*, and *sspH2* were 72.4%, 98.9%, 97.7%, 95.4%, 79.3%, and 89.7%, respectively. *SopE* gene was present in 33 isolates with a detection rate of 37.9%. *SpvB* and *spvR* genes were detected in one isolate, while neither *spvC* nor *pefA* genes were present. All isolates harbored at least six virulence genes, and 62.1% (54/87) isolates were positive to nine or more virulence genes. A total of 20 different virulence gene profiles (VP1~VP20) among the 87 *Salmonella* 4,[5],12:i:- isolates were observed, and VP1 (*invA*-*sseL*-*mgtC*-*siiE*-*sopB*-*gipA*-*gtgB*-*sspH1*-*sspH2*) was the primary one, accounting for 33.3% (29/87), as shown in Table 4 and S3 Fig.

**Table 4:** Virulence gene profiles of the *Salmonella* 4,[5],12:i:- isolates.

Virulence gene profiles	*Salmonella* pathogenicity island genes					Prophage virulence genes					Plasmid virulence genes				No.(%)
	*invA*	*sseL*	*mgtC*	*siiE*	*sopB*	*gipA*	*gtgB*	*sopE*	*sspH1*	*sspH2*	*spvB*	*spvC*	*spvR*	*pefA*	
VP1	+	+	+	+	+	+	+	−	+	+	−	−	−	−	29(33.3)
VP2	+	+	+	+	+	+	+	+	+	+	−	−	−	−	14(16.1)
VP3	+	−	+	+	+	+	+	−	+	+	−	−	−	−	12(13.8)
VP4	+	+	+	+	+	+	+	+	−	−	−	−	−	−	7(8.0)
VP5	+	+	+	+	+	+	+	−	−	+	−	−	−	−	5(5.7)
VP6	+	−	+	+	+	+	+	+	+	+	−	−	−	−	5(5.7)
VP7	+	−	+	+	+	+	+	−	−	+	−	−	−	−	2(2.3)
VP8	+	−	+	+	+	+	+	+	−	+	−	−	−	−	1(1.1)
VP9	+	−	+	+	+	+	−	−	+	+	−	−	−	−	1(1.1)
VP10	+	−	+	+	+	+	−	−	−	+	−	−	−	−	1(1.1)
VP11	+	+	+	+	+	−	+	+	+	+	−	−	−	−	1(1.1)
VP12	+	+	+	+	+	+	+	−	−	−	−	−	−	−	1(1.1)
VP13	+	−	+	+	+	+	+	−	+	+	−	−	+	−	1(1.1)
VP14	+	−	+	+	+	−	+	+	+	+	−	−	−	−	1(1.1)
VP15	+	+	+	+	+	+	+	+	+	−	−	−	−	−	1(1.1)
VP16	+	+	+	+	+	+	+	+	+	+	+	−	−	−	1(1.1)
VP17	+	+	+	+	+	+	+	+	−	+	−	−	−	−	1(1.1)
VP18	+	+	+	+	+	+	−	−	+	+	−	−	−	−	1(1.1)
VP19	+	+	+	+	+	+	−	+	+	+	−	−	−	−	1(1.1)
VP20	+	+	−	+	+	+	+	−	+	+	−	−	−	−	1(1.1)

+ indicates the existence of the gene; − indicates the nonexistence of the genes

### MLST typing

All the 87 *Salmonella* 4,[5],12:i:- isolates were classified into three STs by MLST typing (Fig 2 and S4 Fig). ST34 was the dominant sequence type of *Salmonella* 4,[5],12:i:-, accounting for 97.7% (85/87). The other two STs were ST19 and ST1746, respectively. ST34 and ST1746 were a single-locus variant of ST19, with only one allele locus difference. The dnaN allele was distinct between ST34 and ST19 (dnaN19 replaced dnaN7). The purE allele was distinct between ST1746 and ST19 (purE6 replaced purE5).

## Discussion

*Salmonella* 4,[5],12:i:- has increased significantly in human cases of *Salmonellosis* within the past two decades [10]. In Europe, *Salmonella* 4,[5],12:i:- was the third most common serotype of human *Salmonellosis*, accounting for 7.9% of foodborne disease outbreaks [33]. In recent years, this serotype has been increasing in China, and it has become one of the four most common serotypes causing human *Salmonellosis* [34]. Previous studies suggested that most *Salmonella* 4,[5],12:i:- isolates were from pigs and pork products, while other sources were thought to be rare [33, 35]. Here, the prevalence of *Salmonella* 1,4,[5],12:i:- clinical isolates in Guizhou over six years was higher compared with other regions and countries [16, 34].

Tests of susceptibility to 16 antimicrobial agents showed that isolates exhibited high resistance to sulfamethoxazole, streptomycin, ampicillin, tetracycline, and doxycycline (82.5%~92.0%), which was similar to other studies in China [36, 37], but much higher than that reported in Korea [16] and Japan [38]. Extended-spectrum cephalosporins and fluoroquinolones are the two most crucial antimicrobials for the treatment of invasive and severe infection of *Salmonella*, and the third/fourth-generation cephalosporins are mainly known as "Critically important antimicrobials" [39]. In the present study, we observed 19.5% and 14.9% isolates were resistant to ceftriaxone and cefepime, respectively. In addition, the sensitivity of ciprofloxacin decreased significantly, which may affect the clinical treatment effect. Azithromycin is a significant antibacterial drug with safety and excellent activity in treating *Salmonellosis* [40]. Notably, we found 3.4% *Salmonella* 4,[5],12:i:- isolates were co-resistance to azithromycin, ciprofloxacin, third /fourth-generation cephalosporins, and azithromycin. Carbapenems are atypical β-lactamase antimicrobial with the broadest antimicrobial spectrum, and it is still infrequent in the treatment of *Salmonellosis* [41]. Unfortunately, an imipenem-resistant *Salmonella* 4,[5],12:i:- isolate, collected from a 9-month-old boy in 2015 from Tongren, was found in this study. Our results indicated that the antimicrobial resistance phenomenon of *Salmonella* 4,[5],12:i:- in Guizhou was not optimistic and dynamic monitoring of antimicrobial resistance should be strengthened for this serotype.

Isolates showed a high-level MDR (89.6%) in our study, which was much higher than that of previous studies in China [34], Switzerland [42], and Denmark [35]. Over the last two decades, two primary MDR clones of *Salmonella* 4,[5],12:i:- were recognized as important for public health [43]. One clonal line (European clone) was characterized by chromosomally encoded resistance to ampicillin, streptomycin, sulfonamides, and tetracyclines (ASSuT), and another clonal line (Spanish clone) was characterized by plasmid-encoded resistance to ampicillin, chloramphenicol, sulfonamides, gentamicin, streptomycin, tetracycline, and trimethoprim (ACSuGSTTm) [10, 43]. In our study, 44.8% and 12.6% isolates displayed similar MDR profiles with European and Spanish clones, respectively. Moreover, isolates exhibited more comprehensive MDR profiles. In our study, isolates showed resistance to 13 of the 16 antimicrobial agents, further limiting the selection of antimicrobials for clinical treatment of *Salmonella* 4,[5],12:i:- infection.

Antimicrobial resistance gene detection showed that most isolates harbored *bla*_*TEM-1*_, *strA-strB*, *sul*2 and *tetB* genes regardless of origin. These genes are present in a chromosomal resistance island of *Salmonella* [10]. They are typically associated with the European clone [10, 15], which suggested that the resistant clone of *Salmonella* 4,[5],12:i:- isolates from Guizhou might be related to the European clone. There was a specific correlation between the antimicrobial phenotypes of β-lactamase, phenicols, aminoglycosides, sulfonamides, and tetracyclines with their resistance genotypes. Among the phenicols resistance genes, *cmlAl* and *floR* genes were found to have a moderate and fair correlation with phenotypic resistance of chloramphenicol, respectively. They can be located in large plasmids and transposons of *Salmonella* 4,[5],12:i:-, enabling the transfer of resistance genes between isolates [44]. Among the aminoglycosides resistance genes, the *strA-strB* gene has been found to have moderate correlation with phenotypic resistance of streptomycin. However, the streptomycin phenotype was mainly related to *aph(3’)-Ia*, *aadA1*, and *aadA2* genes in Huang et al [45]. Among the sulfonamides resistance gene, *sul2* gene was found to have fair correlation with phenotypic resistance of sulfaisoxazole. *Sul2* gene was dominant in most sulfonamides resistance mechanisms of *Salmonella* 4,[5],12:i:- and always coexisted with *sul1*, *sul3*, and *dfrA12* genes [44]. Among the tetracyclines resistance gene, *tetB* gene has been correlated with phenotypic resistance of tetracycline and doxycycline. This gene is the most common active efflux gene and ribosomal protective gene for resistance to tetracyclines in *Salmonella* 4,[5],12:i:-.

Significantly, extended-Spectrum β-lactamase (ESBLs) *Salmonella* is considered a severe global public health problem [10]. In this study, *bla*_*TEM-1*_ was the most frequent ESBLs gene consistent with Eastern China [37] and Thailand [46]. It is known that *bla*_*CTX-M*_ is a plasmid-mediated ESBLs enzyme that preferentially hydrolyzes ceftriaxone or cefotaxime, becoming an effective mechanism of Salmonella resistance to broad-spectrum cephalosporin [47]. In our study, the *bla*_*CTX-M*_ gene has been found to have a substantial correlation with the phenotypic resistance of ceftriaxone and cefepime. Five different *bla*_*CTX-M*_ genes were identified, of which *bla*_*CTX-M-55*_ was the most prevalent in Guizhou. The detection rate of *bla*_*OXA-1*_ (5.7%) in this study was lower than that in Eastern China (18.4%) [37], but higher than that reported in Europe (0.21%) [48] and the United States (0.22%) [49]. It is worth noting that a proportion of isolates contained multiple ESBLs genes, which may enhance the adaptability of *Salmonella* 4,[5],12:i:- to cephalosporin drugs, thus affecting clinical treatment outcomes. Therefore, great attention should be paid to these isolates in future resistance monitoring. In our study, several isolates carried antimicrobial resistance genes without showing antimicrobial resistance phenotype. It might be because the drug resistance mechanism of *Salmonella* 4,[5],12:i:- was very complex and some resistance genes were not investigated in this study [50]. A more comprehensive drug resistance mechanism investigation of *Salmonella* 4,[5],12:i:- by whole genome sequencing needs to be performed in our future studies.

The pathogenicity of *Salmonella* involves different virulence genes, which contribute to the invasionand reproduction of *Salmonella* in a complex environment [13]. The *invA*, *sseL*, *mgtC*, *siiE*, and *sopB* genes were highly conserved and were genetic markers for the *Salmonella* pathogenicity island (SPI) in *Salmonella* [51]. The high prevalence of these virulence genes in our study also indicated widespread and highly conserved. The prophage virulence genes *gipA*, *gtgB*, *sspH1*, and *sspH2* were highly prevalent, while the *sopE* gene was present in a few isolates. The *sopE* and *gipA* genes can be transferred by phages, then grow and survive in the Peyer’s patches, which will significantly increase the toxicity of *Salmonella* [9]. By contraries, we found that plasmid virulence genes had shallow detection in *Salmonella* 4,[5],12:i:- isolates. The plasmid virulence *spvC* and *pefA* genes were not detected in all *Salmonella* 4,[5],12:i:- isolates. Similar observations have been recorded in previous studies [34, 52]. The *pefA* gene product facilitates bacterial attachment to host epithelial cells [53]. In contrast, *SpvC*, a phosphothreonine lyase, is an effector protein involved in immune evasion in the early stages of infection and dissemination of the pathogen at the later stages [53]. These virulence genes were more prevalent in *Salmonella* Typhimurium and *Salmonella* Enteritidis isolates but were rare in *Salmonella* 4,[5],12:i:- [34, 54], which indicated that the presence of plasmid virulence in *Salmonella* might be related to specific serotypes. Generally, the plasmid virulence genes of *Salmonella* play a role in systemic infection, but not in the gastrointestinal form [55]. In our study, whether the absence of plasmid virulence genes in *Salmonella* 4,[5],12:i:- isolates were related to the origin of these isolates mainly from feces remains to be further confirmed.

In the last 20 years, the prevalent ST clones of *Salmonella* 4,[5],12:i:- have been changed. ST19 was the main ST clone in the US and Europe during 1991–2016, but the ST34 clone has become increasingly common since 2014 [10, 37]. In our study, all *Salmonella* 4,[5],12:i:- isolates were assigned to three STs and ST34 was the main clone, which was consistent with the European epidemic clone [11, 36]. MLST clustering tree showed that the genetic distance between ST34, ST1746 and ST19 was very close, with only one allele loci difference, indicating that *Salmonella* 4,[5],12:i:- ST19 was likely to be their clonal ancestors. Microevolution between different ST clones isolates remains determined by the Whole-genome sequencing technology and phylogenetic analysis.

## Conclusions

In summary, we characterized the antimicrobial resistance, antimicrobial resistance gene, virulence profiles, and MLST of *Salmonella* 4,[5],12:i:- isolates from 2013 to 2018 in Guizhou, located in the southwest of China. Here, the prevalence of this serotype was at a high level. Isolates showed high rates of resistance to sulfamethoxazole, streptomycin, ampicillin, tetracycline, and doxycycline. A high burden of MDR was observed. Some isolates were co-resistant to ciprofloxacin, third and fourth-generation cephalosporins, and azithromycin had been found. Furthermore, the emergence of carbapenem-resistant and XDR isolates in *Salmonella* 4,[5],12:i:-. It is of great importance to strengthen the drug resistance monitoring of this serotype. Virulence genes and drug resistance genes were carried more frequently. The most common antimicrobial resistance genes were *bla*_*TEM-1*_, *strA-strB*, *sul2* and *tetB*. A certain correlation between the antimicrobial phenotypes and genotypes was found. The examined *Salmonella* 4,[5],12:i:- isolates were mainly ST34. Our findings might be helpful to preliminary understand the characterization of this serotype in Guizhou. Further studies are needed to assess *Salmonella* 4,[5],12:i:- in more detail to better understand the antimicrobial resistance, pathogenicity, and genetic background.

## Supporting information

S1 Fig Identification of Salmonella Typhimurium and *Salmonella* 4,[5],12:i:- isolates by mPCR. (DOCX)

S2 Fig The PCR figures of resistance genes tested in this study. (DOCX)

S3 Fig The PCR figures of virulence genes tested in this study. (DOCX)

S4 Fig The PCR figure of housekeeping genes tested in this study. (DOCX)

S1 Table The fljB and fljB-fljA genes primer and PCR cycling conditions information. (XLSX)

S2 Table Antimicrobial resistance genes primer and PCR cycling conditions information. (XLSX)

S3 Table Virulence genes primer and PCR cycling conditions information. (XLSX)

S4 Table Housekeeping genes primer and PCR cycling conditions information. (XLSX)

S1 Raw images (PDF)
