# Supplementary material for: Highly prevalent MDR, frequently carrying virulence genes and antimicrobial resistance genes in Salmonella enterica serovar 4,[5],12:i:- isolates from Guizhou Province, China
Source: PLoS One. 2022 May 19;17(5):e0266443. doi: 10.1371/journal.pone.0266443 (PMC9119451; doi:10.1371/journal.pone.0266443)
Supplement: S1 Fig — (DOCX) [file pone.0266443.s001.docx]

Supplementary S1 Fig.


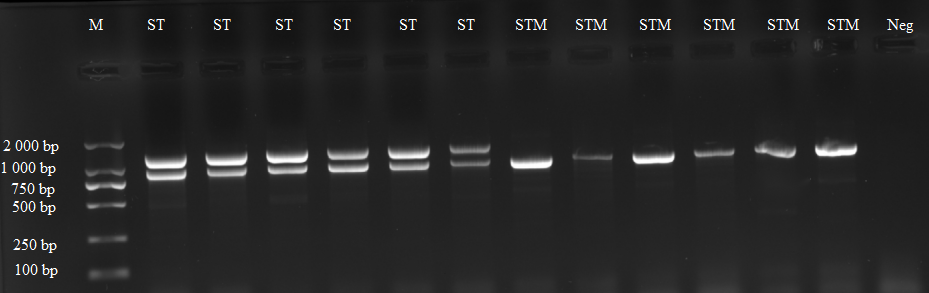


**S1 Fig. Identification of *Salmonella* Typhimurium and *Salmonella* 4,[5],12:i:- isolates by mPCR.** Lane M: DL2 000 DNA Marker. Lanes ST: the representative *Salmonella* Typhimurium isolates produced two amplicons (1 000 bp and 1 389 bp). Lanes STM: the representative *Salmonella* 4,[5],12:i:- isolates produced a single amplicon (1 000 bp). Neg: the negative control (template without DNA).
