# Supplementary material for: Highly prevalent MDR, frequently carrying virulence genes and antimicrobial resistance genes in Salmonella enterica serovar 4,[5],12:i:- isolates from Guizhou Province, China
Source: PLoS One. 2022 May 19;17(5):e0266443. doi: 10.1371/journal.pone.0266443 (PMC9119451; doi:10.1371/journal.pone.0266443)
Supplement: S2 Fig — (DOCX) [file pone.0266443.s002.docx]

Supplementary S2 Fig. The PCR figures of resistance genes tested in this study.


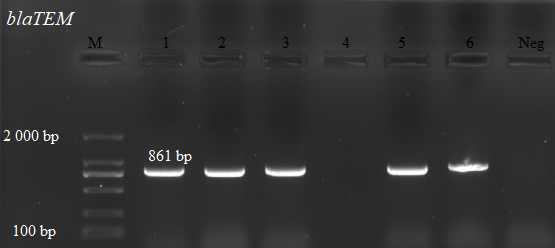


**S2-1 Fig. Electrophoretic pattern of targeting *bla_TEM_* gene.** Lane M: DL2 000 DNA Marker. Lanes 1-6: the specific DNA product (861 bp) amplified from representative isolates of *Salmonella* 4,[5],12:i:-. Neg: the negative control (template without DNA).


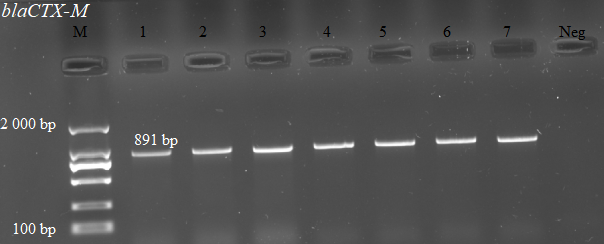


**S2-2 Fig. Electrophoretic pattern of targeting *bla_CTX-M_* gene.** Lane M: DL2 000 DNA Marker. Lanes 1-7: the specific DNA product (891 bp) amplified from representative isolates of *Salmonella* 4,[5],12:i:-. Neg: the negative control (template without DNA).


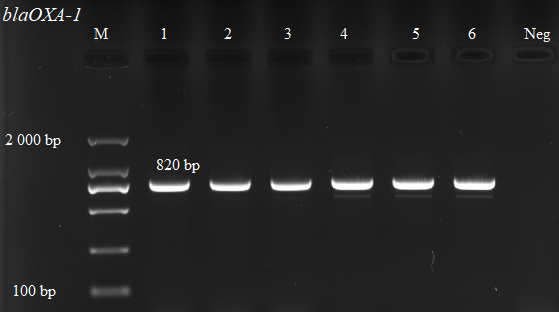


**S2-3 Fig. Electrophoretic pattern of targeting *bla_OXA-1_* gene.** Lane M: DL2 000 DNA Marker. Lanes 1-6: the specific DNA product (820 bp) amplified from representative isolates of *Salmonella* 4,[5],12:i:-. Neg: the negative control (template without DNA).


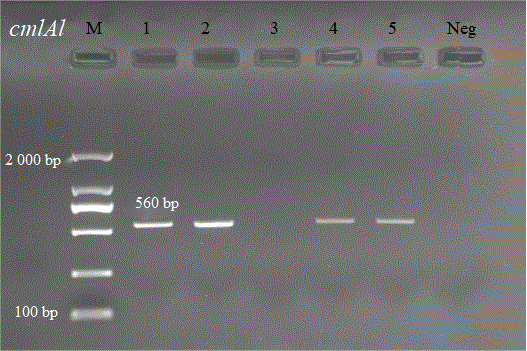


**S2-4 Fig. Electrophoretic pattern of targeting *cmlAl* gene.** Lane M: DL2 000 DNA Marker. Lanes 1-5: the specific DNA product (560 bp) amplified from representative isolates of *Salmonella* 4,[5],12:i:-. Neg: the negative control (template without DNA).


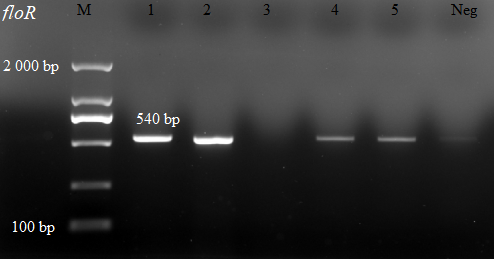


**S2-5 Fig. Electrophoretic pattern of targeting *floR* gene.** Lane M: DL2 000 DNA Marker. Lanes 1-5: the specific DNA product (540 bp) amplified from representative isolates of *Salmonella* 4,[5],12:i:-. Neg: the negative control (template without DNA).


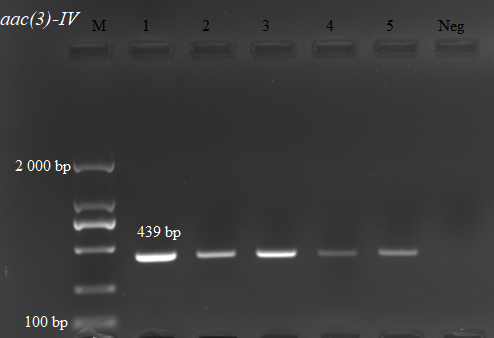


**S2-6 Fig. Electrophoretic pattern of targeting *aac (3)-IV* gene.** Lane M: DL2 000 DNA Marker. Lanes 1-5: the specific DNA product (439 bp) amplified from representative isolates of *Salmonella* 4,[5],12:i:-. Neg: the negative control (template without DNA).


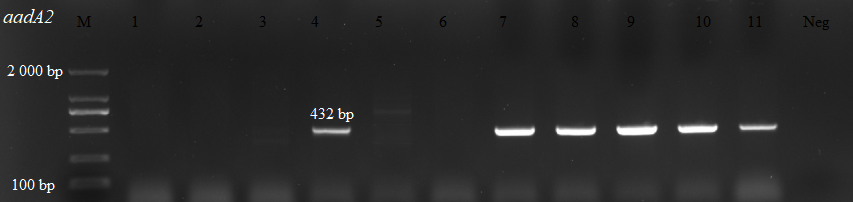


**S2-7 Fig. Electrophoretic pattern of targeting *aadA2* gene.** Lane M: DL2 000 DNA Marker. Lanes 1-11: the specific DNA product (432 bp) amplified from representative isolates of *Salmonella* 4,[5],12:i:-. Neg: the negative control (template without DNA).


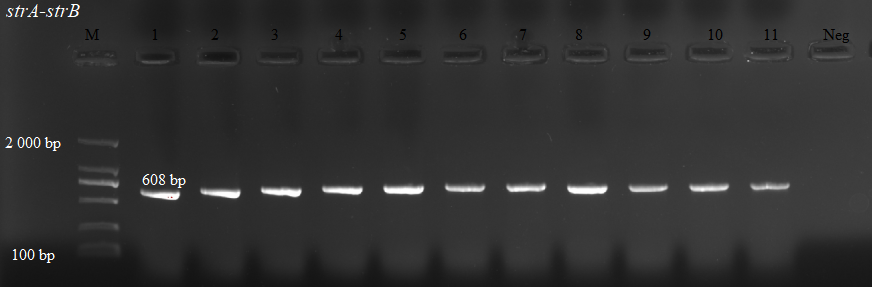


**S2-8 Fig. Electrophoretic pattern of targeting *strA-strB* gene.** Lane M: DL2 000 DNA Marker. Lanes 1-11: the specific DNA product (608 bp) amplified from representative isolates of *Salmonella* 4,[5],12:i:-. Neg: the negative control (template without DNA).


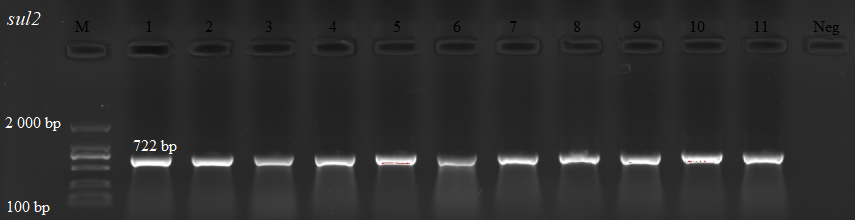


**S2-9 Fig. Electrophoretic pattern of targeting *sul2* gene.** Lane M: DL2 000 DNA Marker. Lanes 1-11: the specific DNA product (722 bp) amplified from representative isolates of *Salmonella* 4,[5],12:i:-. Neg: the negative control (template without DNA).


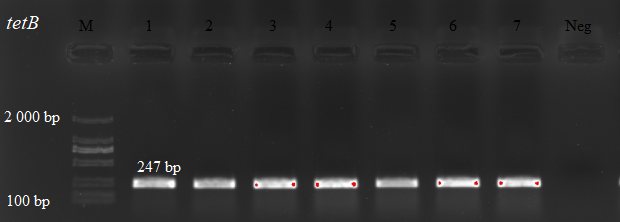


**S2-10 Fig. Electrophoretic pattern of targeting *sul2* gene.** Lane M: DL2 000 DNA Marker. Lanes 1-7: the specific DNA product (247 bp) amplified from representative isolates of *Salmonella* 4,[5],12:i:-. Neg: the negative control (template without DNA).
