# Supplementary material for: Highly prevalent MDR, frequently carrying virulence genes and antimicrobial resistance genes in Salmonella enterica serovar 4,[5],12:i:- isolates from Guizhou Province, China
Source: PLoS One. 2022 May 19;17(5):e0266443. doi: 10.1371/journal.pone.0266443 (PMC9119451; doi:10.1371/journal.pone.0266443)
Supplement: S3 Fig — (DOCX) [file pone.0266443.s003.docx]

Supplementary S3 Fig. The PCR figures of virulence genes tested in this study.


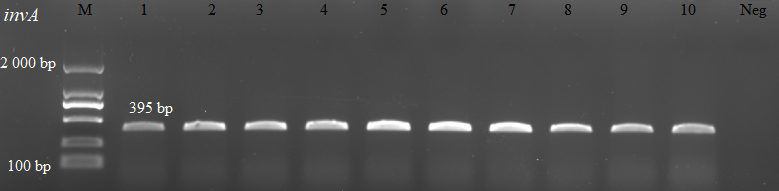


**S3-1 Fig. Electrophoretic pattern of targeting *invA* gene.** Lane M: DL2 000 DNA Marker. Lanes 1-10: the specific DNA product (395 bp) amplified from representative isolates of *Salmonella* 4,[5],12:i:-. Neg: the negative control (template without DNA).


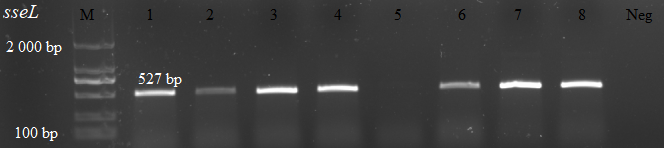


**S3-2 Fig. Electrophoretic pattern of targeting *sseL* gene.** Lane M: DL2 000 DNA Marker. Lanes 1-8: the specific DNA product (527 bp) amplified from representative isolates of *Salmonella* 4,[5],12:i:-. Neg: the negative control (template without DNA).


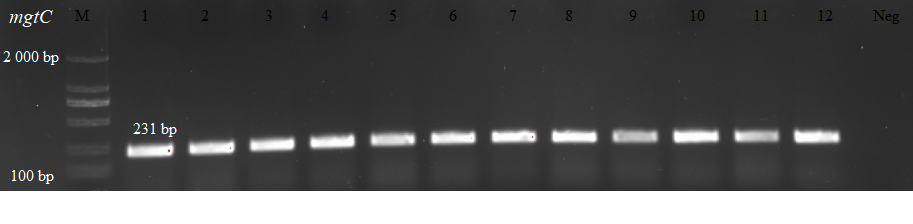


**S3-3 Fig. Electrophoretic pattern of targeting *mgtC* gene.** Lane M: DL2 000 DNA Marker. Lanes 1-12: the specific DNA product (231 bp) amplified from representative isolates of *Salmonella* 4,[5],12:i:-. Neg: the negative control (template without DNA).


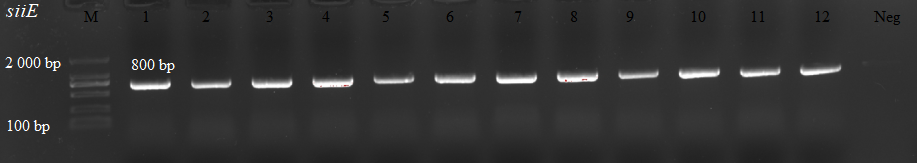


**S3-4 Fig. Electrophoretic pattern of targeting *siiE* gene.** Lane M: DL2 000 DNA Marker. Lanes 1-12: the specific DNA product (800 bp) amplified from representative isolates of *Salmonella* 4,[5],12:i:-. Neg: the negative control (template without DNA).


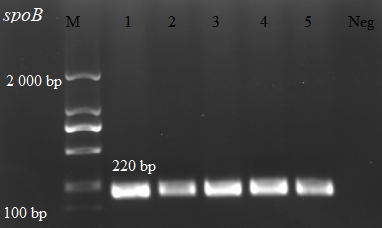


**S3-5 Fig. Electrophoretic pattern of targeting *spoB* gene.** Lane M: DL2 000 DNA Marker. Lanes 1-5: the specific DNA product (220 bp) amplified from representative isolates of *Salmonella* 4,[5],12:i:-. Neg: the negative control (template without DNA).


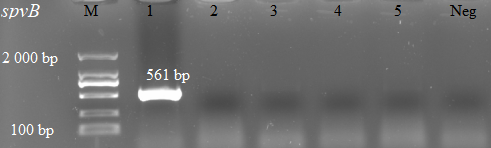


**S3-6 Fig. Electrophoretic pattern of targeting *spvB* gene.** Lane M: DL2 000 DNA Marker. Lanes 1-5: the specific DNA product (561 bp) amplified from representative isolates of *Salmonella* 4,[5],12:i:-. Neg: the negative control (template without DNA).


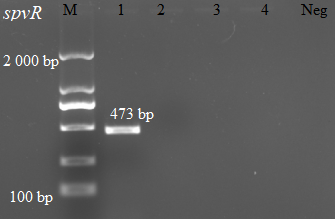


**S3-7 Fig. Electrophoretic pattern of targeting *spvB* gene.** Lane M: DL2 000 DNA Marker. Lanes 1-4: the specific DNA product (473 bp) amplified from representative isolates of *Salmonella* 4,[5],12:i:-. Neg: the negative control (template without DNA).


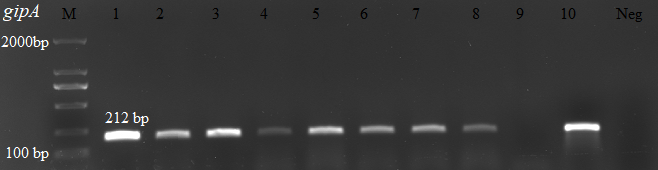


**S3-8 Fig. Electrophoretic pattern of targeting *gipA* gene.** Lane M: DL2 000 DNA Marker. Lanes 1-10: the specific DNA product (212 bp) amplified from representative isolates of *Salmonella* 4,[5],12:i:-. Neg: the negative control (template without DNA).


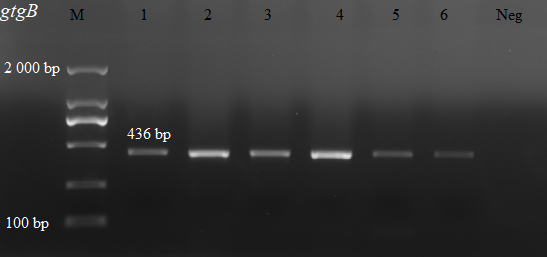


**S3-9 Fig. Electrophoretic pattern of targeting *gtgB* gene.** Lane M: DL2 000 DNA Marker. Lanes 1-6: the specific DNA product (436 bp) amplified from representative isolates of *Salmonella* 4,[5],12:i:-. Neg: the negative control (template without DNA).


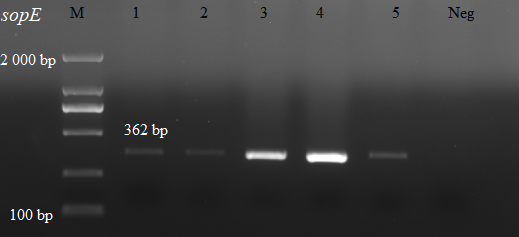


**S3-10 Fig. Electrophoretic pattern of targeting *sopE* gene.** Lane M: DL2 000 DNA Marker. Lanes 1-5: the specific DNA product (362 bp) amplified from representative isolates of *Salmonella* 4,[5],12:i:-. Neg: the negative control (template without DNA).


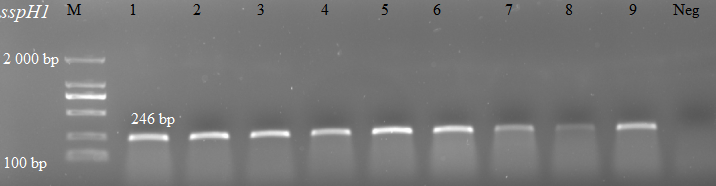


**S3-11 Fig. Electrophoretic pattern of targeting *sspH1* gene.** Lane M: DL2 000 DNA Marker. Lanes 1-9: the specific DNA product (246 bp) amplified from representative isolates of *Salmonella* 4,[5],12:i:-. Neg: the negative control (template without DNA).


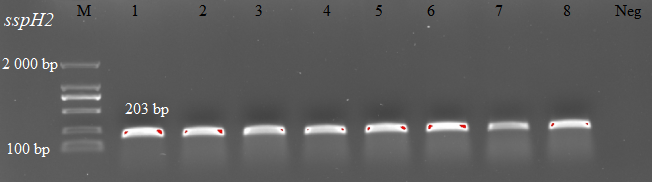


**S3-12 Fig. Electrophoretic pattern of targeting *sspH2* gene.** Lane M: DL2 000 DNA Marker. Lanes 1-8: the specific DNA product (203 bp) amplified from representative isolates of *Salmonella* 4,[5],12:i:-. Neg: the negative control (template without DNA).
