# Supplementary material for: Highly prevalent MDR, frequently carrying virulence genes and antimicrobial resistance genes in Salmonella enterica serovar 4,[5],12:i:- isolates from Guizhou Province, China
Source: PLoS One. 2022 May 19;17(5):e0266443. doi: 10.1371/journal.pone.0266443 (PMC9119451; doi:10.1371/journal.pone.0266443)
Supplement: S4 Fig — (DOCX) [file pone.0266443.s004.docx]

Supplementary S4 Fig. The PCR figure of housekeeping genes tested in this study.


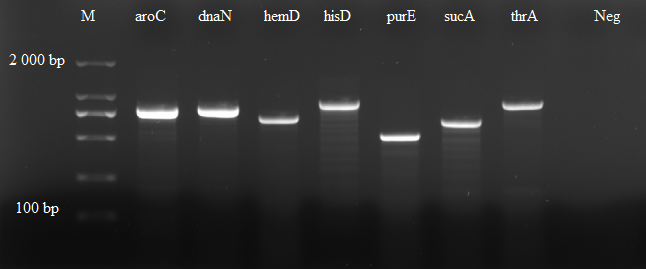


**S4 Fig. Electrophoretic pattern of targeting** housekeeping **gene.** Lane M: DL2 000 DNA Marker. Lanes *aroC* gene (826 bp), Lanes *dnaN* gene (833 bp), Lanes *hemD* gene (666 bp), Lanes *hisD* gene (894 bp), Lanes *purE* gene (510 bp), Lanes *sucA* gene (643 bp), Lanes *thrA* gene (852 bp). Neg: the negative control (template without DNA).
