# Supplementary material for: Highly prevalent MDR, frequently carrying virulence genes and antimicrobial resistance genes in Salmonella enterica serovar 4,[5],12:i:- isolates from Guizhou Province, China
Source: PLoS One. 2022 May 19;17(5):e0266443. doi: 10.1371/journal.pone.0266443 (PMC9119451; doi:10.1371/journal.pone.0266443)
Supplement: S1 Raw images — (PDF) [file pone.0266443.s009.pdf]

Supplementary S1 Fig.

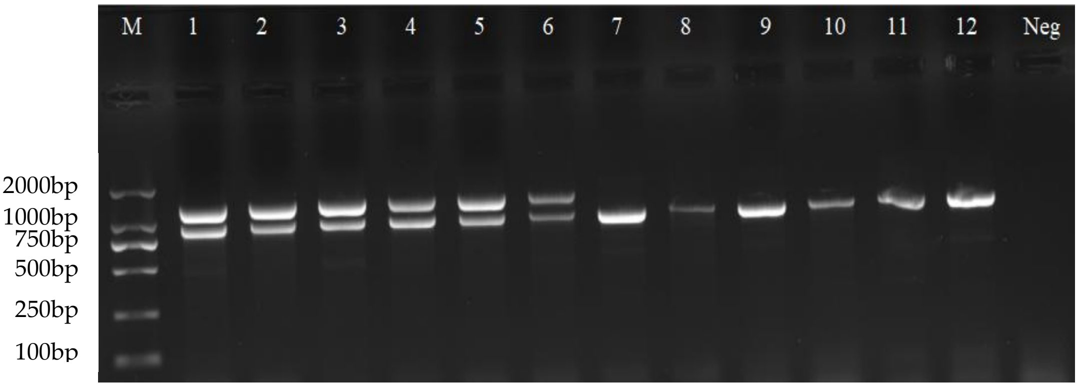

S1 Fig. Identification of *Salmonella* Typhimurium and *Salmonella* 4,[5],12:i:- isolates by mPCR. Lane M: DL2 000 DNA Marker. Lanes 1-6: the representative *Salmonella* Typhimurium isolates produced two amplicons (1 000 bp and 1 389 bp). Lanes 7-12: the representative *Salmonella* 4,[5],12:i:- isolates produced a single amplicon (1 000 bp). Neg: the negative control (template without DNA).

Supplementary S2 Fig. The PCR figures of resistance genes tested in this study.

(1) ***bla***

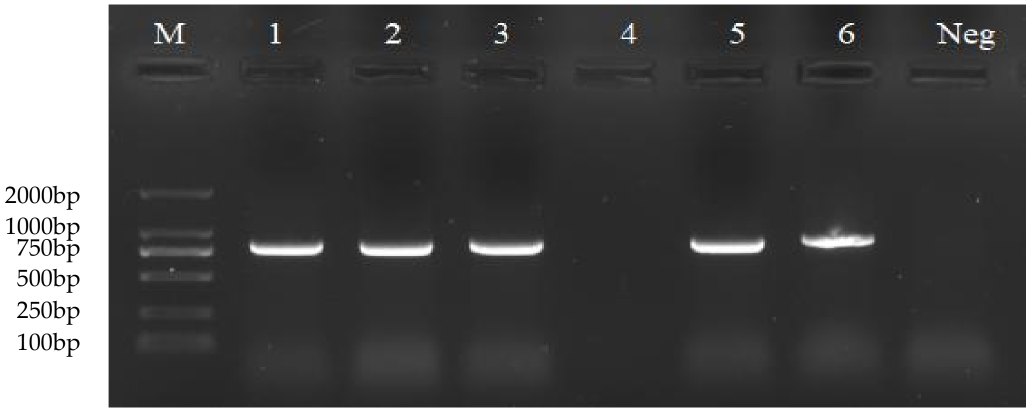

S2-1 Fig. Electrophoretic pattern of targeting bla_{TEM} gene. Lane M: DL2 000 DNA Marker. Lanes 1-6: the specific DNA product (861 bp) amplified from representative isolates of *Salmonella* 4,[5],12:i:-. Neg: the negative control (template without DNA).

(2) $bla_{CTX\text{-}M}$

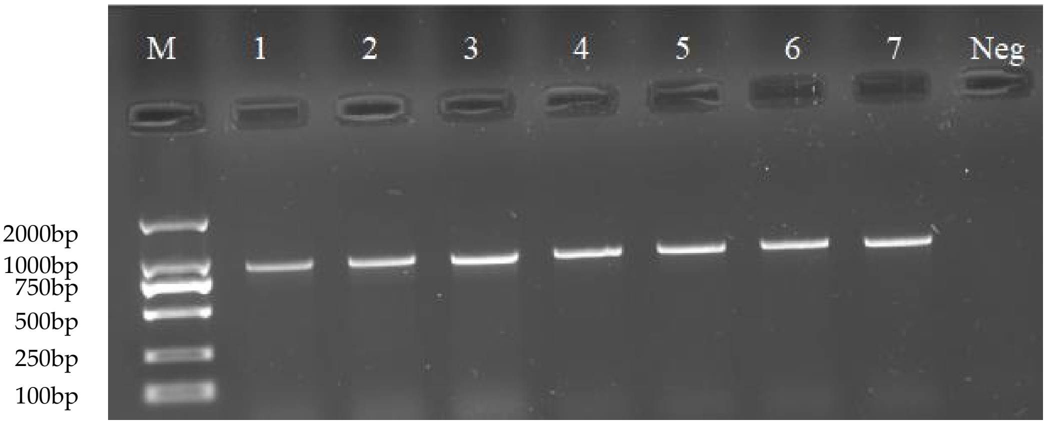

S2-2 Fig. Electrophoretic pattern of targeting $bla_{CTX\text{-}M}$ gene. Lane M: DL2 000 DNA Marker. Lanes 1-7: the specific DNA product (891 bp) amplified from representative isolates of *Salmonella* 4,[5],12:i:-. Neg: the negative control (template without DNA).

(3) ***bla*$_{OXA-1}$**

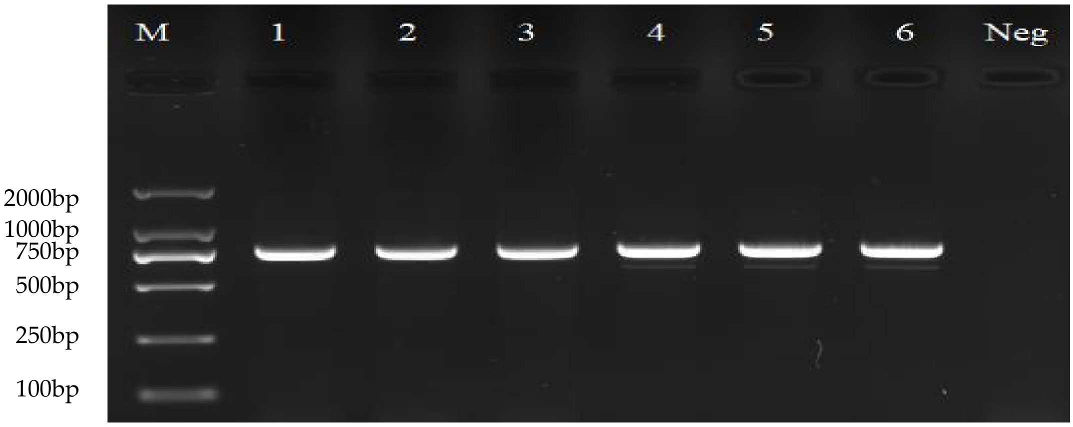

S2-3 Fig. Electrophoretic pattern of targeting *bla*$_{OXA-1}$ gene. Lane M: DL2 000 DNA Marker. Lanes 1-6: the specific DNA product (820 bp) amplified from representative isolates of *Salmonella* 4,[5],12:i:-. Neg: the negative control (template without DNA).

(4) ***cmlAl***

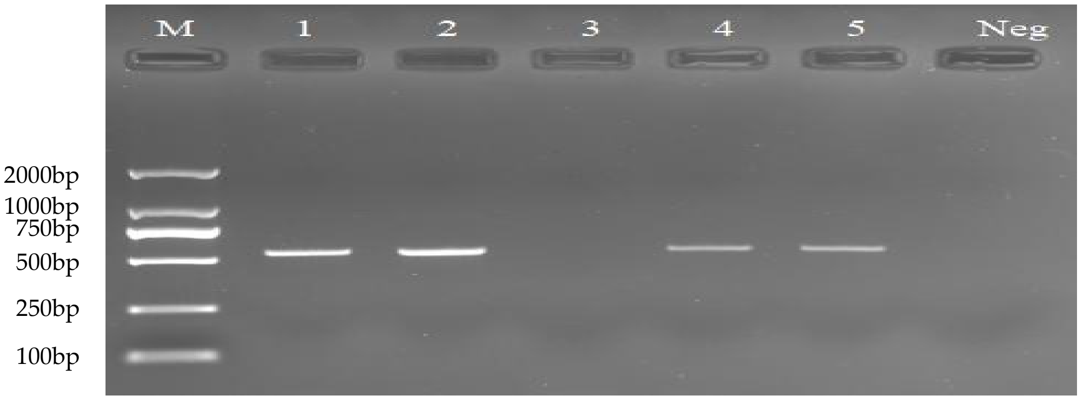

S2-4 Fig. Electrophoretic pattern of targeting *cmlAl* gene. Lane M: DL2 000 DNA Marker. Lanes 1-5: the specific DNA product (560 bp) amplified from representative isolates of *Salmonella* 4,[5],12:i:-. Neg: the negative control (template without DNA).

(5) ***floR***

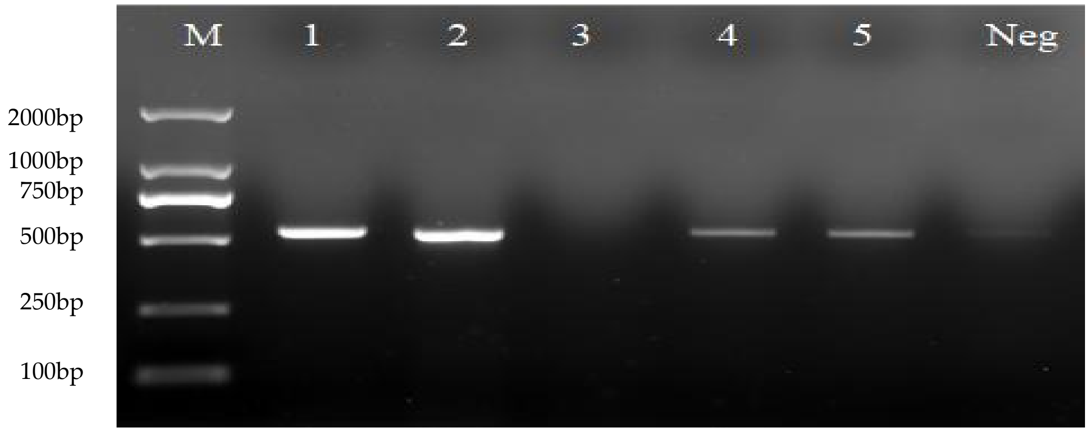

S2-5 Fig. Electrophoretic pattern of targeting *floR* gene. Lane M: DL2 000 DNA Marker. Lanes 1-5: the specific DNA product (540 bp) amplified from representative isolates of *Salmonella* 4,[5],12:i:-. Neg: the negative control (template without DNA).

(6) ***aac (3)-IV***

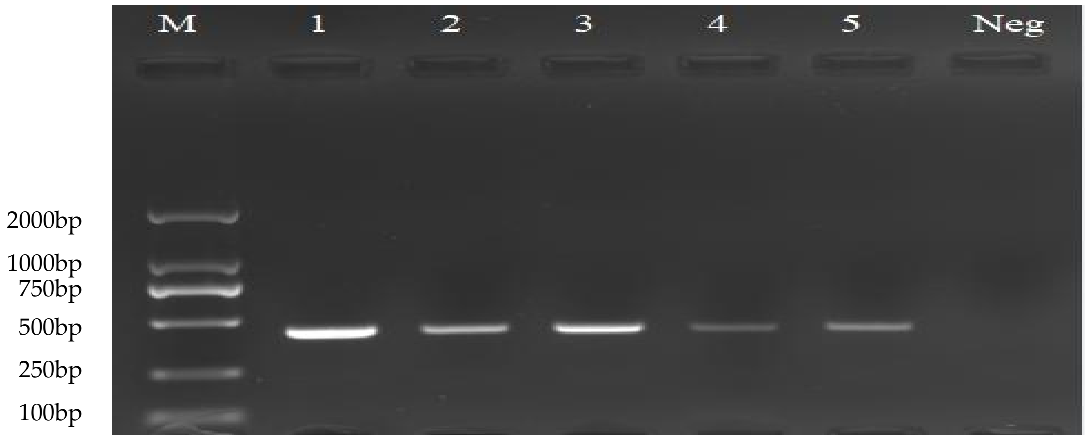

S2-6 Fig. Electrophoretic pattern of targeting *aac (3)-IV* gene. Lane M: DL2 000 DNA Marker. Lanes 1-5: the specific DNA product (439 bp) amplified from representative isolates of *Salmonella* 4,[5],12:i:-. Neg: the negative control (template without DNA).

(7) ***aadA2***

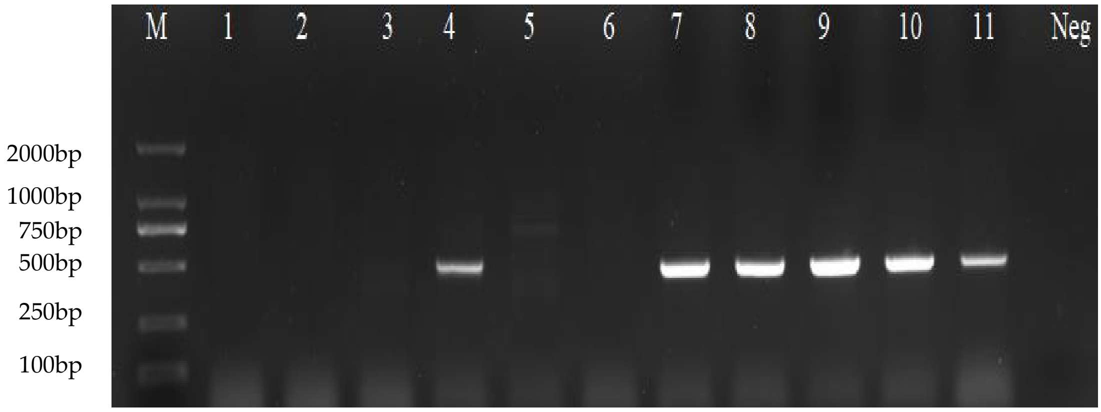

S2-7 Fig. Electrophoretic pattern of targeting *aadA2* gene. Lane M: DL2 000 DNA Marker. Lanes 1-11: the specific DNA product (432 bp) amplified from representative isolates of *Salmonella* 4,[5],12:i:-. Neg: the negative control (template without DNA).

(8) ***strA-strB***

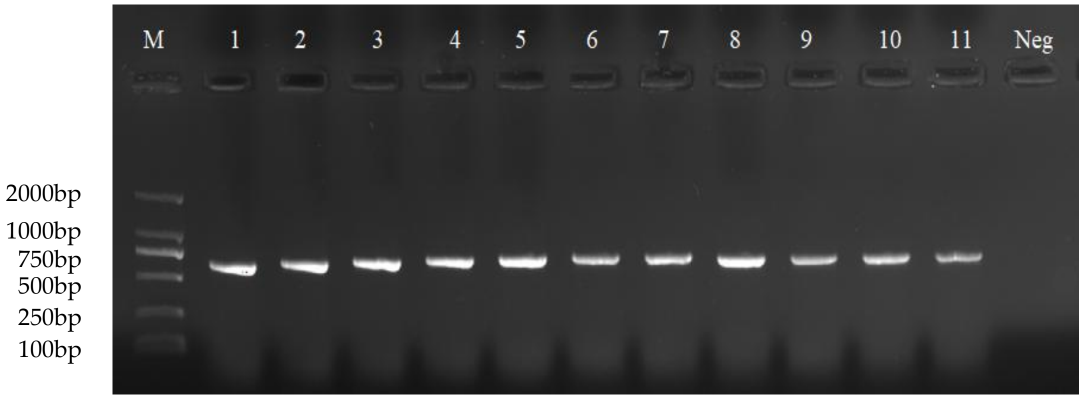

S2-8 Fig. Electrophoretic pattern of targeting *strA-strB* gene. Lane M: DL2 000 DNA Marker. Lanes 1-11: the specific DNA product (608 bp) amplified from representative isolates of *Salmonella* 4,[5],12:i:-. Neg: the negative control (template without DNA).

(9) ***sul2***

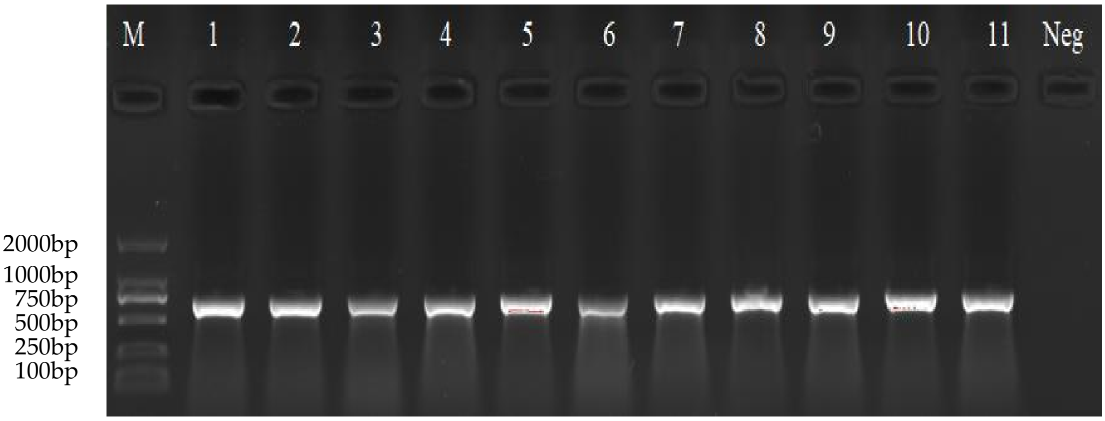

S2-9 Fig. Electrophoretic pattern of targeting *sul2* gene. Lane M: DL2 000 DNA Marker. Lanes 1-11: the specific DNA product (722 bp) amplified from representative isolates of *Salmonella* 4,[5],12:i:-. Neg: the negative control (template without DNA).

(10) ***tetB***

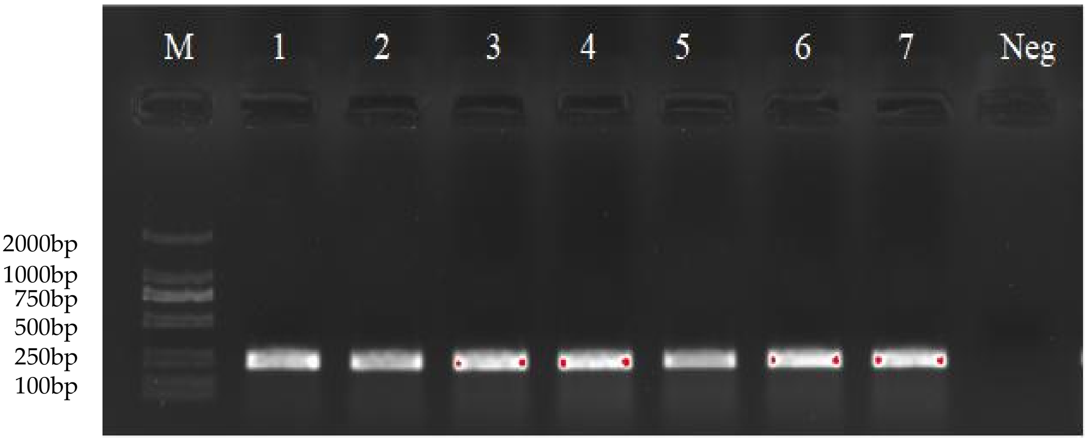

S2-10 Fig. Electrophoretic pattern of targeting *tetB* gene. Lane M: DL2 000 DNA Marker. Lanes 1-7: the specific DNA product (247 bp) amplified from representative isolates of *Salmonella* 4,[5],12:i:-. Neg: the negative control (template without DNA).

Supplementary S3 Fig. The PCR figures of virulence genes tested in this study.

(1) ***invA***

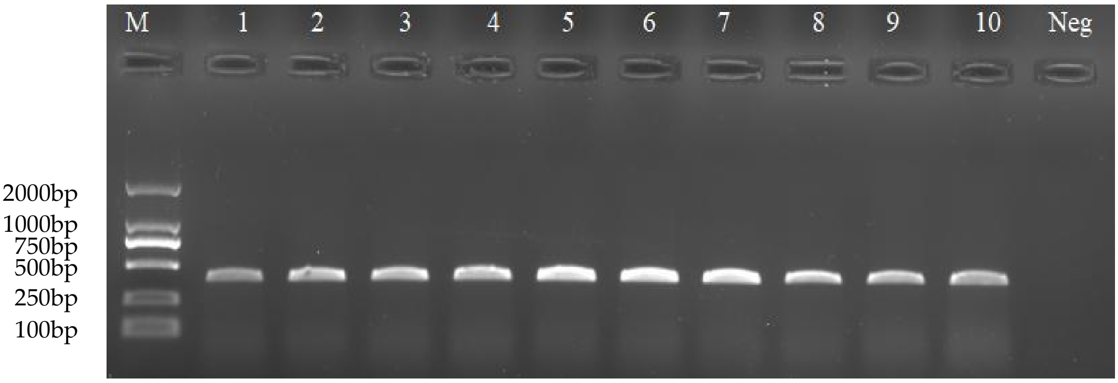

S3-1 Fig. Electrophoretic pattern of targeting *invA* gene. Lane M: DL2 000 DNA Marker. Lanes 1-10: the specific DNA product (395 bp) amplified from representative isolates of *Salmonella* 4,[5],12:i:-. Neg: the negative control (template without DNA).

(2) ***sseL***

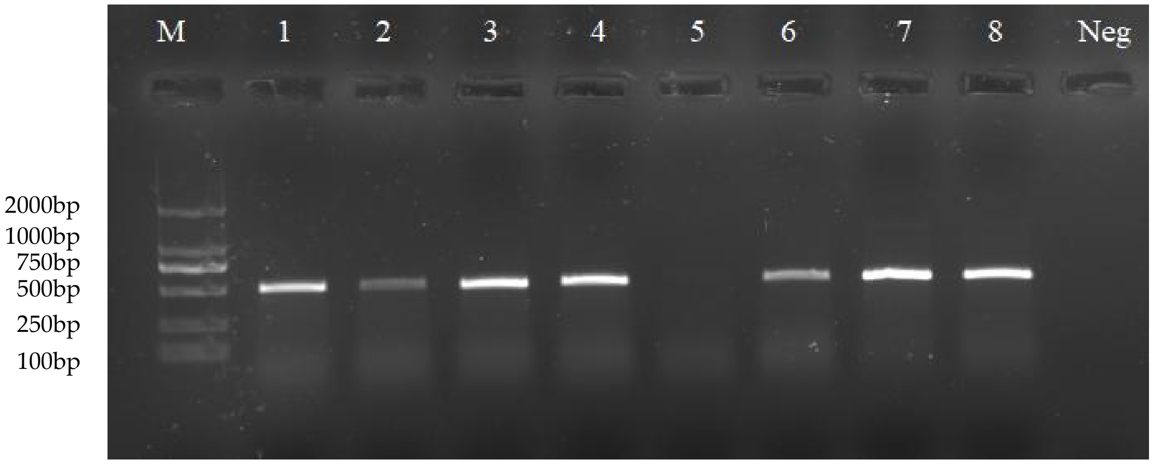

S3-2 Fig. Electrophoretic pattern of targeting *sseL* gene. Lane M: DL2 000 DNA Marker. Lanes 1-8: the specific DNA product (527 bp) amplified from representative isolates of *Salmonella* 4,[5],12:i:-. Neg: the negative control (template without DNA).

(3) *mgtC*

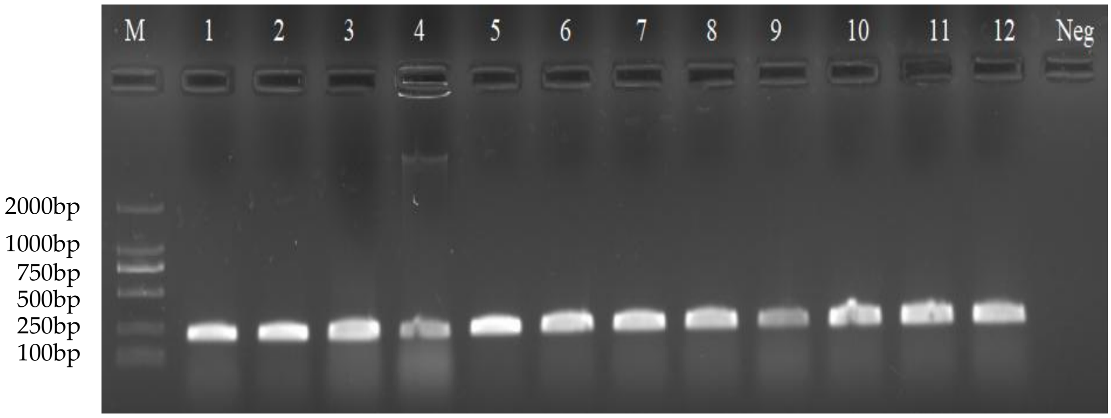

S3-3 Fig. Electrophoretic pattern of targeting *mgtC* gene. Lane M: DL2 000 DNA Marker. Lanes 1-12: the specific DNA product (231 bp) amplified from representative isolates of *Salmonella* 4,[5],12:i:-. Neg: the negative control (template without DNA).

(4) *siiE*

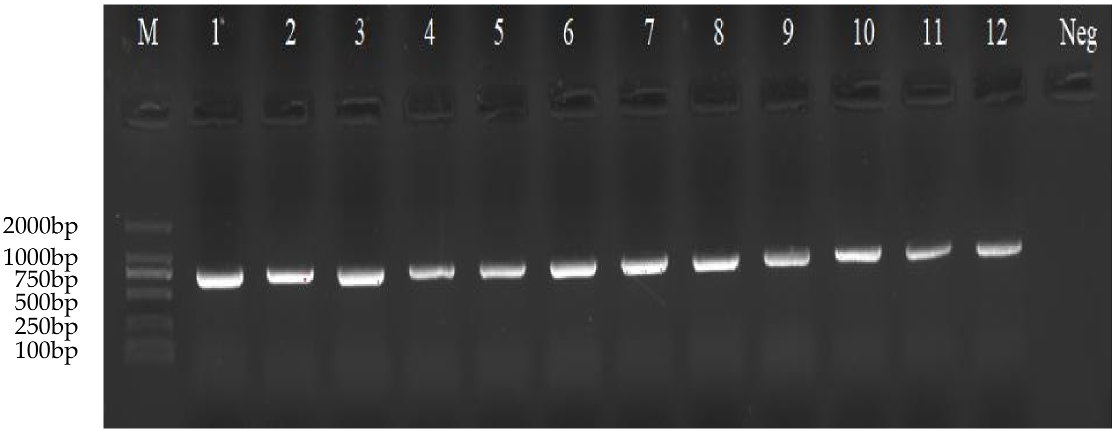

S3-4 Fig. Electrophoretic pattern of targeting *siiE* gene. Lane M: DL2 000 DNA Marker. Lanes 1-12: the specific DNA product (800 bp) amplified from representative isolates of *Salmonella* 4,[5],12:i:-. Neg: the negative control (template without DNA).

(5) ***spoB***

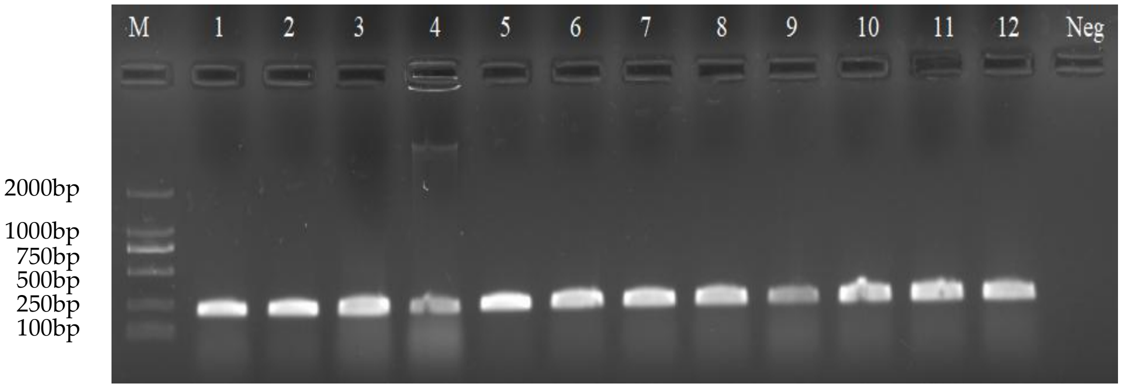

S3-5 Fig. Electrophoretic pattern of targeting *spoB* gene. Lane M: DL2 000 DNA Marker. Lanes 1-12: the specific DNA product (220 bp) amplified from representative isolates of *Salmonella* 4,[5],12:i:-. Neg: the negative control (template without DNA).

(6) ***spvB***

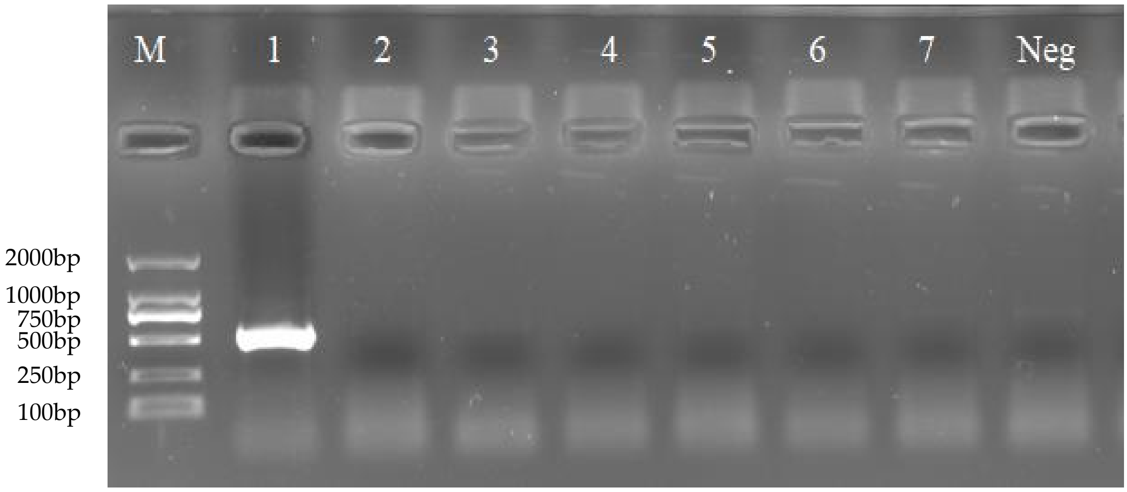

S3-6 Fig. Electrophoretic pattern of targeting *spvB* gene. Lane M: DL2 000 DNA Marker. Lanes 1-7: the specific DNA product (561 bp) amplified from representative isolates of *Salmonella* 4,[5],12:i:-. Neg: the negative control (template without DNA).

(7) *spvR*

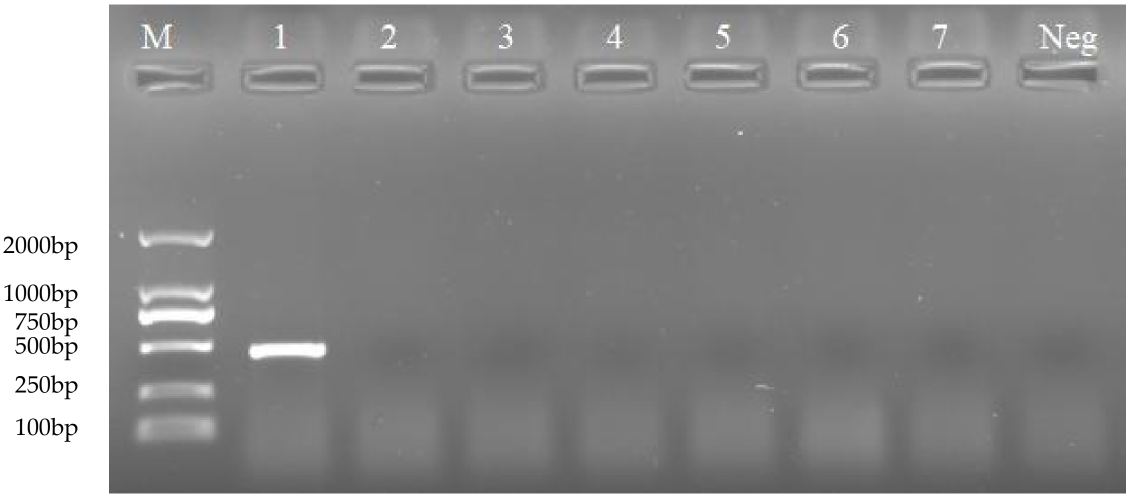

S3-7 Fig. Electrophoretic pattern of targeting *spvR* gene. Lane M: DL2 000 DNA Marker. Lanes 1-7: the specific DNA product (473 bp) amplified from representative isolates of *Salmonella* 4,[5],12:i:-. Neg: the negative control (template without DNA).

(8) *gipA*

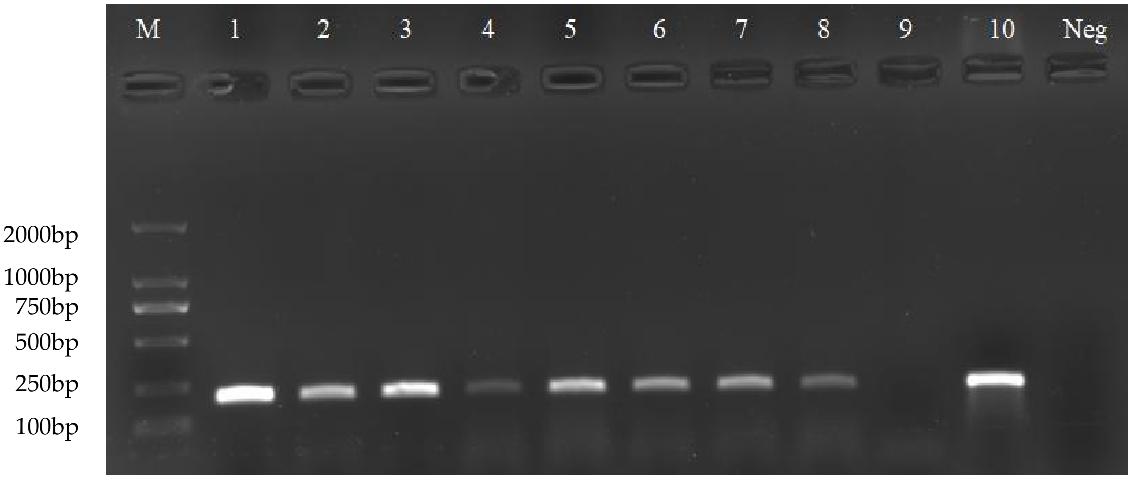

S3-8 Fig. Electrophoretic pattern of targeting *gipA* gene. Lane M: DL2 000 DNA Marker. Lanes 1-10: the specific DNA product (212 bp) amplified from representative isolates of *Salmonella* 4,[5],12:i:-. Neg: the negative control (template without DNA).

(9) *gtgB*

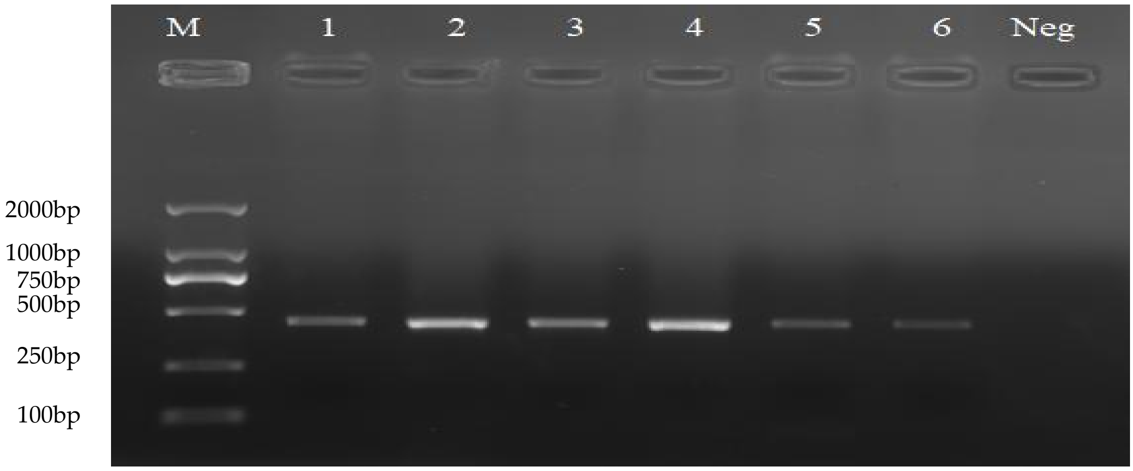

S3-9 Fig. Electrophoretic pattern of targeting *gtgB* gene. Lane M: DL2 000 DNA Marker. Lanes 1-6: the specific DNA product (436 bp) amplified from representative isolates of *Salmonella* 4,[5],12:i:-. Neg: the negative control (template without DNA).

(10) *sopE*

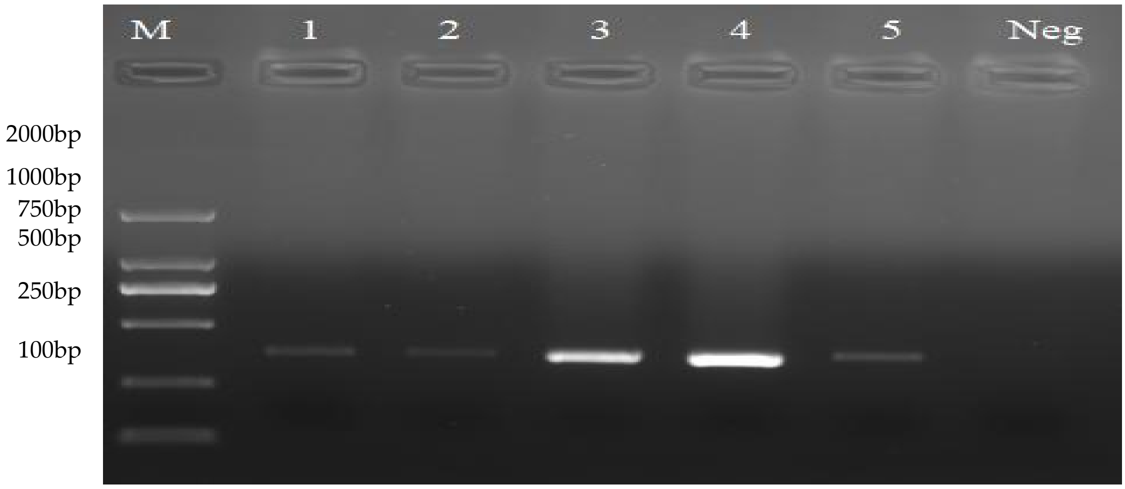

S3-10 Fig. Electrophoretic pattern of targeting *sopE* gene. Lane M: DL2 000 DNA Marker. Lanes 1-5: the specific DNA product (362 bp) amplified from representative isolates of *Salmonella* 4,[5],12:i:-. Neg: the negative control (template without DNA).

(11) ***sspH1***

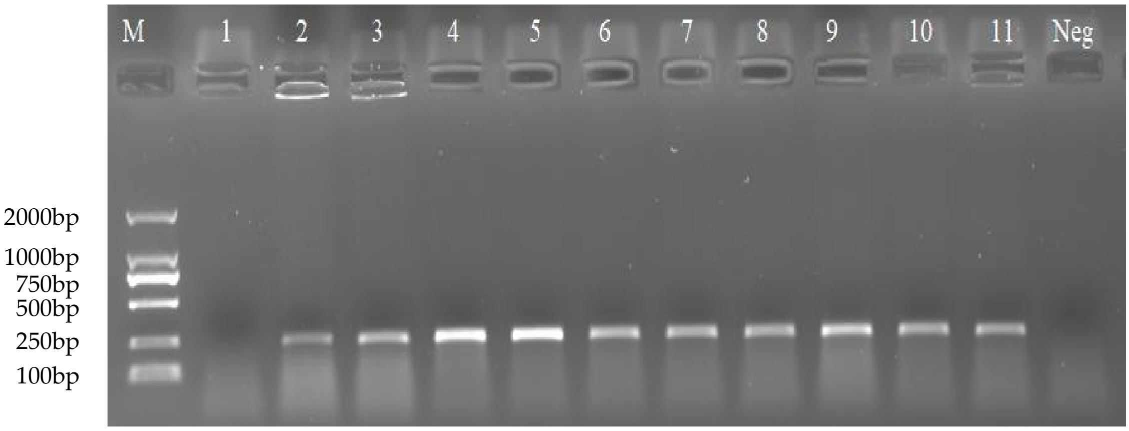

S3-11 Fig. Electrophoretic pattern of targeting *sspH1* gene. Lane M: DL2 000 DNA Marker. Lanes 1-11: the specific DNA product (246 bp) amplified from representative isolates of *Salmonella* 4,[5],12:i:-. Neg: the negative control (template without DNA).

(12) ***sspH2***

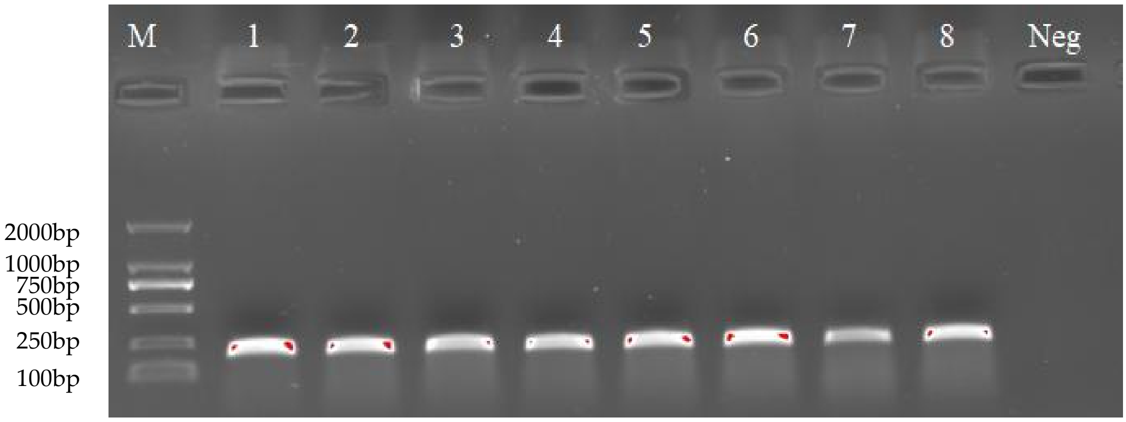

S3-12 Fig. Electrophoretic pattern of targeting *sspH2* gene. Lane M: DL2 000 DNA Marker. Lanes 1-8: the specific DNA product (203 bp) amplified from representative isolates of *Salmonella* 4,[5],12:i:-. Neg: the negative control (template without DNA).

Supplementary S4 Fig. The PCR figure of housekeeping genes tested in this study.

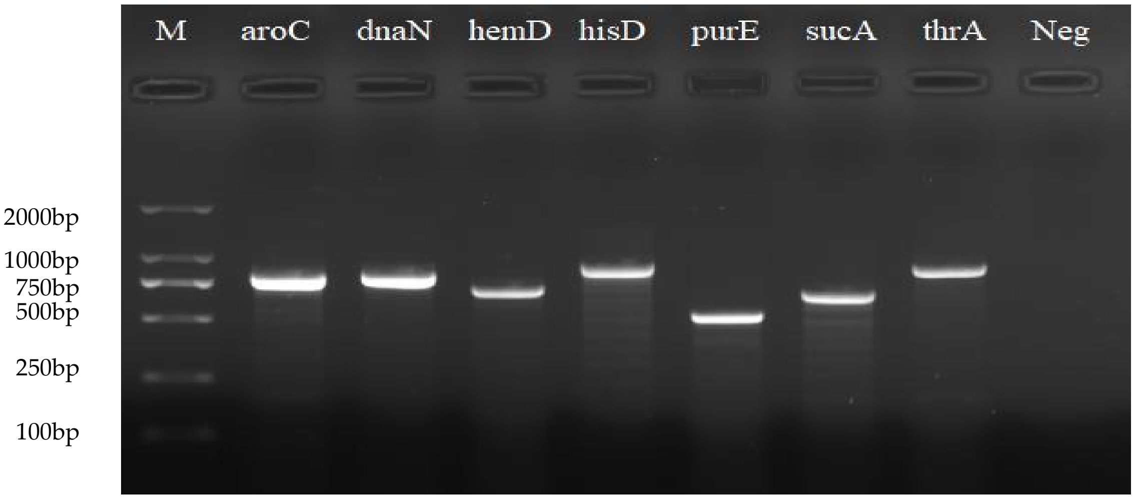

S4 Fig. Electrophoretic pattern of targeting housekeeping gene. Lane M: DL2 000 DNA Marker. Lanes *aroC* gene (826 bp), Lanes *dnaN* gene (833 bp), Lanes *hemD* gene (666 bp), Lanes *hisD* gene (894 bp), Lanes *purE* gene (510 bp), Lanes *sucA* gene (643 bp), Lanes *thrA* gene (852 bp). Neg: the negative control (template without DNA).
